# Developing a global practice-based framework of person-centred care from primary data: a cross-national qualitative study with patients, caregivers and healthcare professionals

**DOI:** 10.1136/bmjgh-2022-008843

**Published:** 2022-07-13

**Authors:** Alessandra Giusti, Panate Pukrittayakamee, Ghadeer Alarja, Lindsay Farrant, Joy Hunter, Olona Mzimkulu, Liz Gwyther, Nokuzola Williams, Kamonporn Wannarit, Lana Abusalem, Sawsan Alajarmeh, Waleed Alrjoub, Lakkana Thongchot, Satit Janwanishstaporn, Adib Edilbi, Ruba Al-Ani, Omar Shamieh, Ping Guo, Kennedy Bashan Nkhoma, Sridhar Venkatapuram, Richard Harding

**Affiliations:** 1 Cicely Saunders Institute of Palliative Care, Policy and Rehabilitation, King's College London, London, UK; 2 King's Global Health Institute, King's College London, London, UK; 3 Faculty of Medicine, Mahidol University, Siriraj Hospital, Bangkok, Thailand; 4 Center for Palliative & Cancer Care in Conflict (CPCCC), King Hussein Cancer Center, Amman, Jordan; 5 School of Public Health and Family Medicine, University of Cape Town, Cape Town, Western Cape, South Africa; 6 College of Medicine, The University of Jordan, Amman, Jordan; 7 School of Nursing, Institute of Clinical Sciences, University of Birmingham, Birmingham, UK

**Keywords:** Health services research, Health policy, Health systems, Qualitative study

## Abstract

**Introduction:**

Person-centred care (PCC) is internationally recognised as a critical component of high-quality healthcare. However, PCC evolved in a few high-income countries and there are limited data exploring this concept across the vast majority of countries which are low- and middle-income. This study aimed to appraise and adapt a PCC model across three serious physical conditions in three middle-income countries and generate an evidence-based framework and recommendations for globally relevant PCC.

**Methods:**

Cross-national, cross-sectional qualitative study. In depth, semistructured interviews conducted with: advanced cancer patients in Jordan (n=50), their caregivers (n=20) and healthcare professionals (HCPs) (n=20); chronic obstructive pulmonary disease patients in South Africa (n=22), their caregivers (n=19) and HCPs (n=22); heart failure patients in Thailand (n=14), their caregivers (n=10) and HCPs (n=12). Data were analysed using framework analysis. Santana *et al*’s PCC model (2018) and Giusti *et al*’s systematic review (2020) were used to construct an a priori coding frame for deductive analysis, with additional inductive coding for coding that did not fit the frame.

**Results:**

The findings both reveal specific practical actions that contribute towards delivering PCC and highlight new cross-national domains of person-centredness: interdependency and collectivism; bringing care into the home and community; equity and non-discrimination; addressing health and illness within the context of limited resources; and workforce well-being.

**Conclusion:**

The data suggest that PCC requires particular structural features of the healthcare system to be in place, such as professional education in PCC values and partnerships with community-based workers. These structures may better enable PCC processes, including tailored information sharing and providing genuine opportunities for patients to do the things that matter to them, such as making informed care decisions and sustaining social relationships. PCC must also accommodate a collectivist perspective and support the well-being of the workforce.

**WHAT IS ALREADY KNOWN ON THIS TOPIC**

- Person-centred care (PCC) is internationally recognised as a dimension of high-quality healthcare, promoted as a core competency of health workers, a key component of primary care and essential to achieving Universal Health Coverage goals.
- PCC is an approach that evolved in high-income countries and there are limited data exploring this concept across low-income and middle-income countries.
- The experience of serious illness is especially aligned with the need for PCC; the complex clinical scenarios surrounding serious illness usually necessitate the involvement of family and/or friends and depend on high-quality communication and joint decision-making to deliver care concordant with patient preferences, and the management of clinical uncertainty.

**WHAT THIS STUDY ADDS**

- Participants highlighted interdependency and collectivism in relation to PCC, including the benefits of social support systems, the value of interpersonal harmony, the interdependence of a person’s and their loved ones’ well-being, and the value of group-based learning, peer-to-peer support and care in the community.
- Participants across all countries and stakeholder groups described limited financial and human resources for health facilities, which contributed towards poor well-being and imposing constraints on a person’s ability to adhere to treatment or advice.
- A widespread participant view was the need to ensure services are accessible to and understandable by persons of all socioeconomic backgrounds and nationalities, and that all patients and caregivers are treated with equal respect by healthcare professionals regardless of financial or other status.

**HOW THIS STUDY MIGHT AFFECT RESEARCH, PRACTICE OR POLICY**

- PCC frameworks must accommodate a collectivist worldview, promoting activities that enable people to support each other and placing focus on community-based and home-based care.
- PCC must consider and go some way to address the social determinants of health and disease, healthcare access and health inequities and should aim to give persons the genuine opportunity to do the things that matter to them.
- PCC must centre around a shared set of human values, including compassion, non-judgementalism, equity and non-discrimination and promote these values in workforce recruitment, training and performance assessment.

## Introduction

 Person-centred care (PCC) is globally recognised as a key component of high-quality healthcare. Since the National Academy of Medicine listed PCC as one the six aims for healthcare quality improvement,^1^ person-centredness has increasingly been advocated by national governments, international organisations and patient and health policy groups. The WHO highlights person‐centredness as a core competency of health workers, a key component of primary care, and essential to achieving Universal Health Coverage goals.^2^,^5^

A variety of terms have been used to denote person-centred approaches, including ‘patient-centredness’ and ‘people-centredness’. Still, person-centred, patient-centred and people-centred care all embody an approach that adopts the perspectives of individuals, families and communities, respects and responds to their needs, values and preferences and sees them as active participants in their own healthcare.^1 6^ Although numerous conceptualisations of PCC have been proposed, few are grounded in empirical research or offer detailed description of PCC practice in diverse contexts. The WHO Global strategy on people-centred and integrated health services recognises that there is not a single model of PCC to be proposed, but that PCC should be context specific, and that each country should generate its own evidence to enable acceptable, feasible practice of PCC.^6^ Nevertheless, PCC is an approach that evolved in high-income country settings, and there are limited data available to model contextually and culturally appropriate PCC in low and middle-income countries (LMICs).^7 8^ Giusti *et al*’s systematic review of the empirical evidence underpinning the concept of PCC for serious illness^7^ indicates a clear need for primary data investigating PCC in a greater diversity of diagnostic groups and settings. Reorienting health systems to become person-centred also has particular salience for LMICs.^9^ Holistic, PCC can help to: reveal and manage socioeconomic barriers to healthcare access and retention^10^,^12^; focus on patient priorities in high-volume health systems with limited per-patient time, reducing repeated admissions and referrals^13 14^ and provide important cost-savings in settings experiencing scarcity of resources.^15 16^ All of these points suggest the need for a global framework of PCC that is relevant and applicable globally, grounded in primary data from diverse country settings.

Person-centred practice is particularly vital in the context of serious illness. The complex clinical scenarios surrounding serious illness usually require the involvement of family members, high-quality communication, management of clinical uncertainty and joint decision-making to deliver care aligned with patient preferences.^17^,^19^ Therefore, the context of serious illness is a means of ‘stress testing’ generalist PCC theory. Furthermore, it is estimated that, out of the 25 million people who died in 2015 experiencing serious health-related suffering, 80% were in LMICs.^20^ Due to ageing populations and improved management of infectious disease, serious health-related suffering and mortality associated with conditions such as cancer, chronic lung disease and heart failure is also rising fastest in LMICs.^20^

The overall aim of this study was to appraise and adapt a prevailing framework of PCC across three serious health conditions in three middle-income countries, and to generate evidence-based recommendations for relevant and acceptable PCC, with potential for local adaptation. The specific objectives were (1) to determine patient, caregiver and healthcare professional (HCP) views on the meaning, feasibility and acceptability of delivering PCC in their settings and (2) to appraise and adapt an empirically underpinned practice-based framework and recommendations to strengthen health systems through PCC, that takes account of socioeconomic, cultural and disease-specific differences.

## Methods

### Design

This was a cross-national, cross-sectional, qualitative study, which used semistructured interviews and framework analysis and is reported in accordance with the consolidated criteria for reporting qualitative research guidelines.^21^ The study focused on advanced cancer, heart failure and chronic obstructive pulmonary disease (COPD); three serious physical illnesses with high prevalence in LMICs, clinical uncertainty and prior evidence of high multidimensional needs.^14 22 23^

### Setting

Data were collected in three countries: Jordan, South Africa and Thailand (online supplemental appendix A summarises key country characteristics). To increase transferability of findings and global applicability of the framework, a range of countries were selected that varied on multiple dimensions: WHO region, spoken languages, dominant religion, population size, percentage of population living below national poverty line and healthcare provision landscape. The choice of condition for each country reflected high local prevalence and morbidity. Due to logistical and time constraints, only one diagnostic group could be focused on in each country. It was decided that selecting a different diagnostic group in each country would allow greater variation of experiences, values and expectations to be captured and enable a resulting PCC framework with greater depth and applicability.

Participants were recruited from one specialist non-governmental centre and one generalist public hospital in Amman, Jordan, one primary care district hospital and four community health centres in Cape Town, South Africa and one public hospital based in Bangkok, Thailand.

### Sampling and recruitment

The study sample consisted of patients, informal caregivers and HCPs. Clinicians at each site approached patient and caregiver participants regarding the study. Those who expressed interest in participation were provided with more detailed information by a local research assistant. Eligible HCPs were introduced to the study by their facility manager, who provided an information sheet and contact details for the local study research assistant. Written consent was obtained from all participants.

Eligibility criteria for patient participants were: aged 18 or over; having physical and psychological capacity to participate; receiving care from a study site; living with advanced cancer (in Jordan), COPD (in South Africa) or stage III or IV heart failure (in Thailand). Eligible informal caregivers were defined as ‘unpaid, informal providers of one or more physical, social, practical and emotional tasks. In terms of their relationship to the patient, they may be a friend, partner, ex-partner, sibling, parent, child or other blood or non-blood relative’.^24^ Eligible HCPs included any healthcare staff who had worked at a study site for at least 6 months providing direct care to advanced cancer, COPD or heart failure patients, respective to site. Eligible participants were required to be able to communicate in either Arabic or English for the Jordan-based interviews, isiXhosa, Afrikaans or English for the South Africa-based interviews, and Thai or English for the interviews conducted in Thailand.

Patients were sampled purposively to achieve heterogeneity in age, gender, country of origin and primary concurrent treatment in Amman, age, gender, marital status and education level in Cape Town, and age, gender, nationality, stage of heart failure, socioeconomic status and primary concurrent treatment in Bangkok. Caregiver participants at all sites were purposively sampled by age, gender and relationship to patient, and HCPs were purposively sampled by age, gender, professional role and years of experience.

### Data collection

Semistructured topic guides (online supplemental appendix B) were developed by AG in collaboration with on-site research assistants (GA, LA, WA, JH, OM, LF, PP) and colleagues working at King’s College London (PG, KBN, RH, SV) and according to PCC literature. The topic guides were translated into Arabic, isiXhosa, Afrikaans and Thai by translators and checked by the local research assistants.

In-depth semistructured interviews with demographic data collection were conducted face-to-face at the study sites during a routine outpatient visit or inpatient admission. In Thailand, participants were also given the option of participating by telephone due to COVID-19 restrictions. Data were collected in Jordan between October 2018 and June 2020, in South Africa between May and December 2019, and in Thailand between July 2020 and April 2021.

Interviews based in Jordan were conducted by GA, LS and WA, who were all employed as researchers and instructors by the King Hussein Cancer Centre and all hold considerable research training and experience. Participants based in South Africa were interviewed by JH (MPhil Palliative Medicine) or OM (MSc Public Health), who both hold previous experience in healthcare research and qualitative interviewing. In Thailand, all interviews were conducted by PP, a practicing psychiatrist holding an MSc in Palliative Care. All interviewers are nationals of the country in which they conducted interviews and participated in a qualitative research training workshop led by AG.

Interviews with patients and caregivers comprised of open questions centring around the interviewee’s main needs, values, priorities and concerns, whether/how those needs were being met, how they would describe the care received and how they would like care to be delivered. The topic guide was adapted for HCPs to include questions focusing on their current practice; their views on willingness to reorient practice to a person-centred approach; anticipated challenges and benefits and their training and support needs.

Data collection continued until it was collaboratively decided by AG and each on-site research team that sufficient information power was reached.^25^ Interviews were digitally audio recorded, transcribed verbatim, translated into English where necessary, and pseudonymised (see online supplemental appendix C for further details of transcription and translation process). A ‘reflexivity log’ (online supplemental appendix D) was completed by interviewers following each interview to record contextual factors, emergent themes, reflections and inform assessment of information power. In particular, interviewers were encouraged to reflect on how their characteristics, values and communication may have been affecting the data they were collecting, and to potentially adjust these where necessary and possible. Written reflections were later discussed in team meetings with AG and considered when each transcript was coded and at later stages of the analysis process.

### Analysis

Data were analysed using framework analysis, combining deductive and inductive approaches and using NVivoPro software to manage the data. Santana *et al*’s PCC model^26^ and Giusti *et al*’s^7^ review of data underpinning the concept of PCC for serious illness were used to construct an a priori coding frame (online supplemental figure A) for deductive data analysis, with additional inductive coding for data that did not fit the a priori frame. The PCC model developed by Santana and colleagues^26^ (hereafter referred to as Santana model) was selected to create a priori code as it provides comprehensive, practical guidance for implementation of PCC, explicitly linking this guidance to the Donabedian model^27^ for assessing healthcare quality by classifying PCC domains into the categories of ‘Structure,’ ‘Process’ and ‘Outcome’. The Santana model has broad applicability and was generated through a narrative review and synthesis of recommendations and best practice from implementation case studies and preexisting frameworks.

The thematic coding framework was constructed collaboratively, drawing on local teams’ knowledge and views throughout. First, the research team in South Africa (JH, OM, LF) participated in a qualitative data analysis workshop led by AG. Each team member (and AG) then individually coded a subselection of the Cape Town interview transcripts (three patient, three caregiver and three HCP interviews), before reconvening to compare coding and develop a coding frame. The coding frame that developed comprised: preselected a priori codes consisting of Santana *et al* PCC model domains,^26^ a priori codes derived from the results of a previously conducted systematic review,^7^ and inductive codes derived by content-related open coding. AG then coded all the remaining transcripts from South Africa using this agreed coding frame. This process was then repeated with the teams based in Jordan and Thailand, with the coding frame forming the basis for the next team’s deductive coding. The single resulting coding frame was repeatedly discussed and recoded to ensure consistency and validity. This highly collaborative process for data analysis enhanced dependability, strengthened the analysis and resulting theory and aided cultural sensitivity. Ideas, hypotheses and decisions were noted in NVivo memos to serve as an audit trail and enhance confirmability. AG indexed and sorted all interview transcripts, created a framework matrix for each broad coding frame category, and led mapping and interpretation of the data.

**Figure 1:**
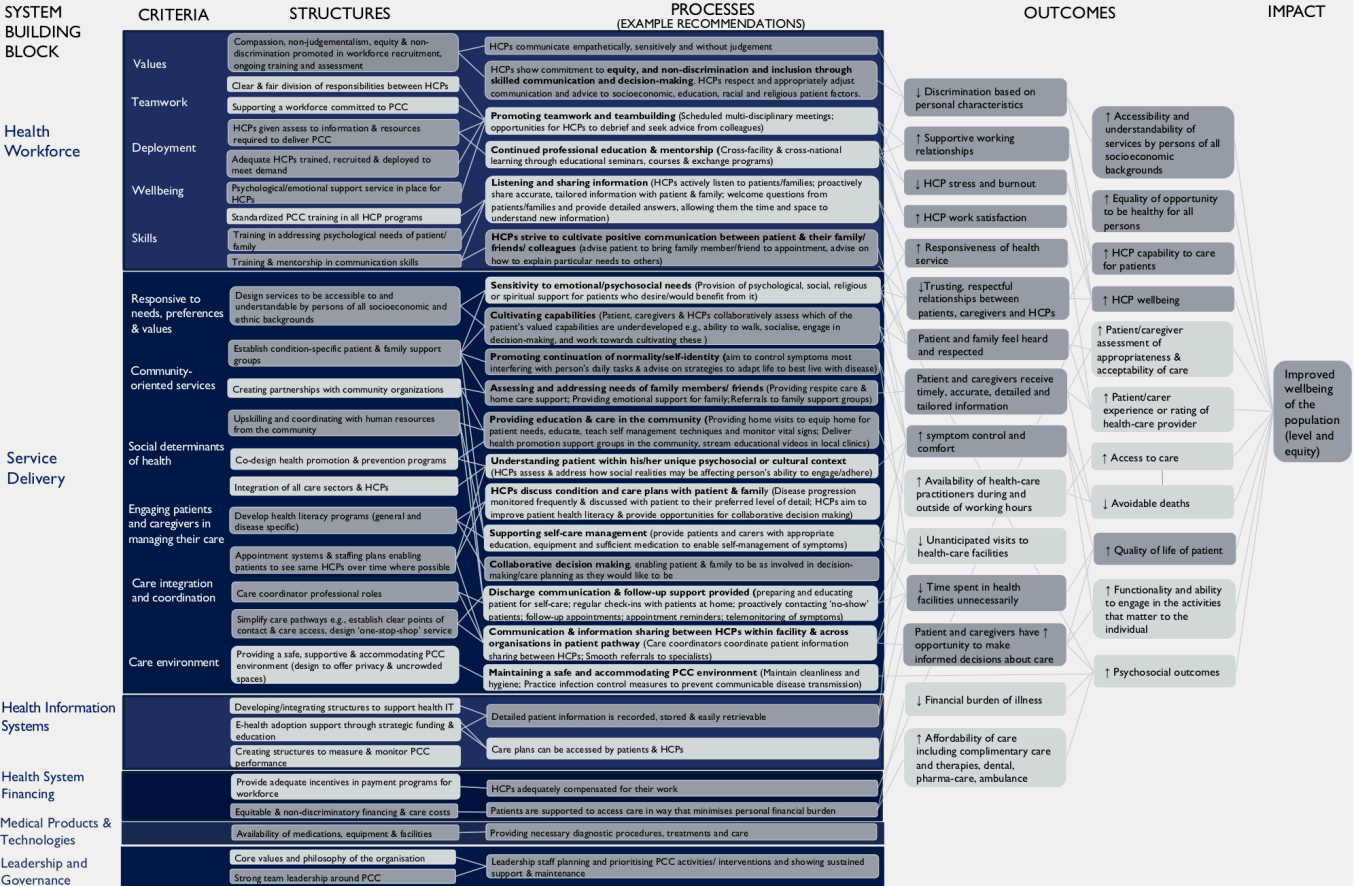
Empirically underpinned systems-oriented framework and recommendations for the practice of person-centred care (Santana *et al* model domains presented in light grey boxes; domains derived from study data through inductive reasoning presented in dark grey boxes). HCP, healthcare professional; PCC, person-centred care.

Key findings were mapped into a framework of PCC (figure 1), with deductively and inductively derived components presented as structures, processes and outcomes of PCC,^27^ and organised by WHO building blocks for strengthening health systems.^28^

Patient and public involvement was not conducted as part of this study.

## Results

### Participants

A total of n=189 participants were recruited (see table 1) between October 2018 and April 2021 and comprised n=50 advanced cancer patients, n=20 caregivers and n=20 HCP in Jordan, n=22 patients with COPD, n=19 caregivers and n=22 HCPs in South Africa and n=14 heart failure patients, n=10 caregivers and n=12 HPCs in Thailand. Purposive sampling parameters were achieved. The mean average duration of interviews was 46 min. The total recruitment rate was 44%. All interviews were conducted face-to-face in the health facility, except for n=1 interview conducted by telephone in Thailand.

**Table 1:** Participant characteristics (n=189) (for full table of participant characteristics see online supplemental table 1)

Jordan					
Patient participants	N=50	Caregiver participants	N=20	HCP participants	N=20
Gender (male/female)	20/30	Gender (male/female)	7/13	Gender (male/female)	13/7
Age (years)		Age (years)		Age (years)	
Mean average (SD)	53.8 (11.8)	Mean average (SD)	41.9 (12.4)	Mean average (SD)	36.5 (8.1)
Range	26–75	Range	19–67	Range	24–55
Nationality		Nationality		Professional role	
Jordanian	28	Jordanian	13	Doctor	9
Syrian	11	Syrian	3	Nurse	11
Libyan	4	Libyan	1		
Iraqi	4	Palestinian	2		
Palestinian	2	Yemeni	1		
Yemeni	1				
**South Africa**					
Patient participants	N=22	Caregiver participants	N=19	HCP participants	N=22
Gender (male/female)	16/6	Gender (male/female)	2/17	Gender (male/female)	9/13
Age (years)		Age (years)		Age (years)	
Mean average (SD)	57.5 (11.6)	Mean average (SD)	45.2 (14.0)	Mean average (SD)	41.5 (9.3)
Range	38–77	Range	22–69	Range	26–55
Education level		Education level		Education level	
Primary school	2	No education	1	Doctor	12
Secondary school	16	Primary school	2	Nurse	5
Tertiary education	1	Secondary	15	Pharmacist	2
Postgraduate	1	College/university	1	Pharmacy manager	1
Post matric	2			Operations manager	1
				Clinical manager	1
**Thailand**					
Patient participants	N=14	Caregiver participants	N=10	HCP participants	N=12
Gender (male/female)	11/3	Gender (male/female)	1/9	Gender (male/female)	1/11
Age (years)		Age (years)		Age (years)	
Mean average (SD)	54 (14.8)	Mean average (SD)	50.4 (8.2)	Mean average (SD)	28.7 (8.5)
Range	22–81	Range	35–62	Range	20–43
Education level		Education level		Professional role	
Primary school	2	Junior high school	1	Nurse	7
Junior high school	1	Senior high school	1	Practical nurse	5
Senior high school	1	Bachelor’s degree	5		
Bachelor’s degree	6	Higher than bachelor’s degree	2		
Higher than bachelor’s degree	2	Vocational diploma	1		
Vocational certificate /diploma	2				

HCP, Healthcare professionals.

### Findings

#### Data aligning with Santana model’s structure, process and outcome domains

##### Structures

The interview data revealed specific practical actions that populate the Santana model domains (italic text) for practical application (see table 2). Regarding structural components, a dominant view among all participant stakeholder groups in all countries was the need to promote a supportive PCC work environment that ‘encourages teamwork and teambuilding’. Specifically, this included fair division of labour and responsibilities and positive, amicable communication between staff in different roles (quote 1). HCPs discussed the importance of supportive relationships at work and the value of being able to debrief and seek advice from colleagues (quotes 2, 3).

**Table 2:** Illustrative participant quotations for Santana *et al* PCC model themes (for full table of Santana model themes and illustrative quotations see online supplemental table 2)

Santana *et al* themes corresponding to illustrative quotations	Number	Illustrative quotations
**Encourage teamwork and teambuilding**	1	By dividing the roles between the medical staff everything would be easy. I mean it is hard to bear all the responsibility by yourself only, you need help from others. HCP16, HCP, Male, 35, Jordan
	2	With some staff we are able to discuss, what does it mean, how you feel about this, what has frustrated you, between the doctors and the nurses and the doctors and other doctors and then between the doctors and specialists. I think there are supportive relationships, I think there could be ways in which they could be more supportive potentially but I am not sure exactly how or how that would look. Maybe create another space where we can discuss. PROF01-005, HCP, Male, 32, South Africa
	3	We have collaborated for a long time; we get along well including the doctor, nurse, and pharmacist and we understand each other. Having a personal bond helps us collaborate well. 3010, HCP, Female, 43, Thailand
**Prioritising the safety and security of patients and staff**	4	The reason why I am attending here is because that other hospital is situated in gang land. The gang comes in there when there is a shooting there and they come after the one that they shot, then you must all be hiding. Now, how on earth can you feel free or safe to go like operate like that? PAT01-005, Patient, Male, 69, South Africa
	5	They are at risk of infections in the hospital so that is why if you have a separate suite that was just for the COPDs and asthmas, you don’t have the TBs floating in there and making them ill. PROF01-002, HCP, Male, 45, South Africa
	6	It is not crowded unlike the outpatient unit of the internal medicine department…there are so many people standing around us. It is such a bad situation, especially during the COVID-19 pandemic situation. We need to do physical distancing but we can’t since every patient has their relatives with them. 2005, Caregiver, Female, 50, Thailand
**Cultivating communication and** **sharing information**	7	Nurse X and Nurse Y are great and helpful. You should see the conversation between me and X, she always urges me to ask questions and comforts me. PAL0044, Patient, Female, 50, Jordan
	8	I like to ask a lot of questions and he always tells me I am doing fine. I know he doesn’t want me to ask questions… Even if he clarifies he does it minimally: “Your condition is stable”. PKH0020, Patient, Female, 42, Jordan
	9	If you ask for something, they will advise you…They will not push you aside. They will always have an answer if you do ask a question, which is always in a good way. PAT07-003, Patient, Male, 73, South Africa
	10	I like the doctor gives me opportunities to ask questions. 1014, Patient, Male, 41, Thailand
	11	The patient also might not know the important things they need to know or the options they have. By giving them the information, you’re putting the ball in their court. HCP06, HCP, Female, 31, Jordan
	12	The doctors don't even really speaks so a lot. And sometimes my mommy is in such a lot of pain and you can see it’s just, just sitting here and she doesn't even say a word. CAR03-002, Caregiver, Male, 28, South Africa
	13	The patients required a doctor’s diagnosis explanation in relation to their present and future symptoms, and treatment methods. They expected the doctor’s explanation without asking as they were worried that they would be reprimanded by the doctor if they did. 3010, HCP, Female, 43, Thailand
	14	I want the doctor to talk to me about my condition, I don’t like it when he’s vague. I want to know what I can eat, what stage I am at, what my current situation is…The doctor stops by in the morning, asks me how I am doing and then leaves. PAL0003, Patient, Female, 47, Jordan
	15	As doctors we don’t spend enough time with our patients explaining to them this is what is wrong with you, this is what you can expect, these are the improvements that you can expect, this is what happens when you are unwell. I think if patients understand what it is that is wrong with them it will help them to cope with their illness better. PROF01-003, HCP, Female, 31, South Africa
	16	If I know how my symptoms will progress and what the treatment procedures are, it makes me feel good…I don’t have the knowledge, so I get curious and worried. 1013, Patient, Male, 50, Thailand
	17	We need to improve our approach in terms of asking people of how much information they want. HCP10, HCP, Female, 40, Jordan
	18	The doctor should ask first if the patient wants to know or not. If he wants to know, the doctor can gradually give him information. If he does not want to know and the doctor tells him, he will be very worried. 1010, Patient, Female, 60, Thailand
**Shared decision-making**	19	It should be a joint decision…It is possible that the doctors want to do something that I don’t approve of. When it was time for my sixth cycle, I told Dr X that it is making me ill…so he told it was ok if I didn’t want to take it. PKH0023, Patient, Male, 55, Jordan
	20	Most of the time they will listen, they will respond, we will make a decision together. CAR04-002, Caregiver, Female, 26, South Africa
	21	The patient and I greatly took part in decision making; the doctor gave us information and let us opt in or out…It’s good because the surgery has risks and the patient will feel that she chooses to accept the risks… Collaboration is likely to bring about the best treatment method. 2010, Caregiver, Female, 52, Thailand
	22	I am not a doctor…I believe the doctor should just inform the patient about the plan because the doctor knows what’s best for the patient. People tend to get emotional and they might freak out and decide against chemo just because they don’t want to lose their hair or lose weight. PKH0021, Patient, Female, 26, Jordan
	23	I tend to just tell the patient, “We are going to keep the medication same, everything looks good, are you happy?” And then most of the time they will say, “No it is fine,” because they feel fine. PROF04-002, HCP, Female, 29, South Africa
	24	I would like the doctors to decide because they can do it better. They have studied medicine, so they know what to do. I am just a patient, and I don’t know anything. It’s better to let doctors decide. 1012, Patient, Male, 72, Thailand
	25	In the western world, the patient comes into the doctor’s office with more knowledge on their condition than the doctor does. They come in ready to discuss whatever they want to know. The patients here come in and put the decision in the hands of their family, which their family puts in the hands of the doctor…you share the facts and what you know with the family so they can make a decision and they just tell you to do whatever you feel is right. HCP18, HCP, Male, 54, Jordan
	26	If the patient doesn’t know anything, we’d have to make the decisions with the family. HCP7, HCP, Female, 27, Jordan
**Understanding the patient within his/her unique psychosocial or cultural context**	27	It is God who will cure me, I always ask him to strengthen me, and that’s when I feel that he loves me, I like to think that he is putting me through this as a form or redemption from my sins or to get me closer to him. PAL040, Patient, Female, 43, Jordan
	28	I think in terms of spiritually when people start realising that they are not going to live very long. And as health professionals we don’t often have those conversations although in palliative care we should. PROF07-002, HCP, Female, 49, South Africa
	29	The patient pays homage to a Buddha image and prays before bed every night; she then feels better and calmer. 2010, Caregiver, Female, 52, Thailand
	30	I seem to be opposed to religion of all kinds because in my mind’s eye I see it as brainwashing. PAT01-003, Patient, Male, 61, South Africa
	31	Religion is not the patient’s spiritual anchor. His spiritual anchor is his mother who always encourages him. 2003, Caregiver, Female, 43, Thailand
**Care access**	32	It’s hard to get the ambulance or when she has a regular follow up the ambulance won’t come, her husband puts her in the car, the nurse carries her and I hold the chair and we cram in the car, she gets in a lot of pain. CKH0015, Caregiver, Female, 67, Jordan
	33	My transport is my main thing I need. So, I just leave it like that because sometimes, maybe I get a COPD attack in the night and then I phone the ambulance, they do not come. Like the other time I was waiting for eight hours, till the morning and I had to ask my neighbour to come bring me here. PAT03-001, Patient, Male, 42, South Africa
	34	Many patients travelled from remote provinces…those whose appointments had been scheduled for many consecutive days had to stay in Bangkok for several nights. When they stayed in an unfamiliar accommodation, they couldn’t sleep thus making them have fatigue and higher blood pressure. 3012, HCP, Female, 20, Thailand
**Financial burden and affordability of care**	35	This cycle is probably the last one I am gonna take because I can’t afford any more. I can barely get my children bread…I went to UNHCR and they told me funding was stopped especially for those who came in 2014. PAL0037, Patient, Female, 45, Jordan
	36	Finances are one of the main aspects we try to secure for these patients because this also affects their mental health. We see many depressed patients as a result of financial struggles. HCP1, HCP, Male, 41, Jordan
	37	Basically I need to have that R200 bucks as a spare in case I am gonna need to Taxify or Uber or I am going to need to have for an emergency…So, what you will find now it puts a strain on what do we cut from grocery list? CAR04-002, Caregiver, Female, 26, South Africa
	38	The doctor is considering if an additional medication should be prescribed but I’d have to pay an additional 1500 baht per month…It is a diabetes medication which would improve my condition… it would be nice but I’m making up my mind. 1007, Patient, Male, 60, Thailand
**Patient reported outcome measures (PROMs**)	39	Even if I still have the disease, I want to wake up in the morning, make coffee, I don’t have to cook, I can fix myself something simple, move around the house. I want to reduce the pain so I can be around (my family) again. PKH0011, Patient, Female, 44, Jordan
	40	This disease…it takes away your self will and it does it in an insidious way in that no matter how determined you might be, you can’t physically do x, y or z because you can’t breathe, and if you can’t breathe you can’t do anything. PAT01-001, Patient, Male, 67, South Africa
	41	The patients want to get better steadily until they can go about their normal daily life and go back to work. At the beginning of treatment, the patients get very worried but later after continuous treatment, they would see positive results in which their condition would improve if they took good care of themselves. Even though they wouldn’t completely recover, they got better steadily till they could live their normal daily life and work. They then looked happier. 3008, HCP, Female, 42, Thailand

HCP, healthcare professional.

Aligning with another structural domain from the Santana model, patients, caregivers and HCPs raised the need for facilities to *prioritise the safety and security of patients and staff*. Some patients, particularly those based in Cape Town, reported feeling unsafe at facilities, or on journeys to and from facilities due to high local levels of violent gang crime (quote 4). Safety was also raised in relation to communicable diseases, such as Tuberculosis and COVID-19, and the need to reduce risk of transmission within healthcare facilities (quotes 5, 6).

Some structural components of the Santana model were left unpopulated by data from this study, such as domain *S1b*. *Establishing operational definition of PCC* and domain *E-health adoption support*.

##### Processes

The data strongly aligned with many of the Santana model’s Process dimensions. Relating to *cultivating communication* and *sharing information*, patients and caregivers across all study sites wished to feel welcome to raise topics, ask questions freely, receive detailed answers to their questions and be given the time and space to understand new information (quotes 7–10). HCPs in all settings raised the importance of being proactive in sharing information, as patients may not be aware of all the information that would be useful to them or may not feel comfortable or well enough to ask questions (quotes 11–13). Patient, caregivers and HCPs also described the need for frequently monitoring disease progression and discussing this in detail with the patient (quotes 14–16). However, some patients also expressed the view that negative information or poor prognosis should not be forced on a person if they do not wish to be told (quotes 17, 18).

Participants further supported the need for *shared decision-making* between patient, caregivers and HCPs. However, views on the role and weight each party should hold in the decision-making process were divided. One prominent view was that treatment plan decisions should be made collaboratively, with HCPs contributing their clinical knowledge, patients contributing their lived experience and patients making the final informed decision (quotes 19–21). A contrasting patient preference across countries was to be informed, have options and possible consequences explained, have their preferences considered, but to ultimately not hold the responsibility and ‘burden’ of taking a decision. These patients and caregivers felt that key treatment decisions should be left in the hands of HCPs for reasons including: trusting that ‘the doctor knows better’; believing that they lacked the knowledge to make informed decisions and believing that fear, vulnerability and financial issues may affect their ability to make rational decisions (quotes 22–24). HCPs in all three countries also described the challenge of involving patients in decision-making due to a lack of basic health literacy, lack of desire for involvement and due to a tendency for family members to ask for prognosis not to be disclosed to the patient (quotes 25, 26).

*Understanding the patient within his/her unique psychosocial or cultural context* was also a theme further specified by the data. In particular, many interviewees across countries held strong religious or spiritual beliefs and described ways in which these shaped a person’s perception of illness, such as placing trust in God or fate, or feeling concern that illness may be a punishment from God (quotes 27, 28). Religious faith and practice were also described as a significant source of comfort to persons living with serious illness and provision of spiritual support was deemed to be beneficial to well-being (quote 29). At the same time, some non-religious patient and caregiver participants did not want religious ideologies to influence their care and instead considered family members or good humour to be their spiritual anchor (quotes 30, 31).

##### Outcomes

Patients, caregivers and HCPs also emphasised the Santana model domain**—***care access*—describing long distances to travel to healthcare facilities, lack of transportation and difficulties in travelling to facilities due to poor mobility (quotes 32–34). A widespread barrier to care access was also the *financial burden* of serious illness, including both poor *affordability of care*, transportation and accommodation costs when seeking care and the financial challenges resulting from an inability to work (quotes 35–38). These financial burdens often left patients unable to receive active treatment and/or impacted patient and family members’ mental well-being (quotes 36–38).

While no study participants reported the use of *Patient Reported Outcome Measures* *(PROMs)*, patients and caregivers commonly described the importance of *quality of life, symptoms, functionality* and *psychosocial outcomes*. Participants often spoke of symptom control, comfort and ability to engage in their daily lives as their ultimate priority, even over slowing disease progression. Symptom control was seen as important because of its ability to enable patients to do and be the things that mattered to them, such as regaining the ability to walk, pray, socialise and converse with friends and family, perform daily household tasks and work professionally (quotes 39–41). Poor functionality was, in turn, described as affecting the person’s mental health and willpower (quote 40).

### Data aligning with PCC domains derived from Giusti *et al* systematic review^7^

#### Structures

The study data also provided specific, operationalisable details about the meanings of PCC domains derived from systematic review^7^ (table 3). In line with the domain *Structuring service organisation to enable continuity of care and patient* navigation, participants highlighted the value of having an allocated care coordinator to schedule and remind patients of appointments, direct patients through the healthcare facility, provide information to the patient and coordinate patient information sharing between HCPs (quotes 42, 43). Participants across all countries and stakeholder groups also suggested the need to *simplify care pathways* by establishing clear points of contact and care access, providing a ‘one-stop-shop’ service where possible, scheduling appointments across departments conveniently, and building smooth referral pathways (quotes 44–47). Patient and caregivers also expressed a preference for seeing the same doctor or specialist over time, suggesting the need for appointment systems and staffing plans structured to allow this. Patients proposed that this would better allow a trusting relationship to develop, avoid the need for patients to repeat their health history and better enable HCPs to give tailored information (quotes 48–50). Some patients suggested that seeing a different HCP at each appointment was unproblematic so long as detailed patient information was stored and accessible by each HCP (quote 51).

**Table 3:** Illustrative participant quotations for themes derived from Giusti *et al*. systematic review^7^

PCC domains derived from systematic review	Number	Illustrative quotations
Structuring service organisation to enable continuity of care and patient navigation	42	It’s so important that we have a coordinator because it’s important for the patient to understand the whole situation from the second they’ve been referred to palliative care…We need to let the patient know that we’re going to focus on their symptoms, set a care plan, follow up with them, and provide home care if it’s needed. We are also the link between the patient, their family, the doctor, the hospital, and the pharmacy. HCP12, HCP, Female, 28, Jordan
	43	I think when you determine that the patient has COPD…you need to allocate in a specific uhm…caregiver, mentor…I don’t know quite what the word is, in banking it would be a relationship banker…and I don’t know that that person needs to be a qualified doctor, in fact probably not. But there needs to be somebody who can liaise with them, the doctors, but at the same time is going to be prepared to listen to you. And in fact, have more time to listen to you. PAT01-001, Patient, Male, 67, South Africa
Simplification of care pathways to ease patient navigation	44	If patients can access health care coverage at another hospital, we would advise the patients on how to transfer the coverage from the affiliated hospital to here. The affiliated hospital would allow a transfer of coverage for three months. After three months, we have to write up a document for the patient to bring to the affiliated hospital so that the hospital transfers the coverage to here…The affiliated hospital should allow for more than three months of health care *coverage. 3009, HCP, Female, 21, Thailand*
	45	When you call them on the phone, the first one picking up should be the one to tell you what to do, rather than transferring you from one person to another. I can’t book an appointment; it is a very tiring process. PKH0049, Patient, Female, 56, Jordan
	46	It is better like…When you meet the doctor, you just meet the doctor and then everything you get it from the doctor. And not go to the pharmacy. PAT04-002, Patient, Male, 38, South Africa
	47	Like today after I finished my meeting with the doctor, they let me know where I should go next…They can communicate clearly. Sometimes when we go to a government office, we may not know who to contact and what to do. But here everything is very clear. 1003, Patient, Male, 54, Thailand
Appointment system structured to allow patients to see same HCPs over time	48	Unfortunately, she would book me appointments and at each appointment I would be seen by a different doctor…I don’t know how they could figure out my two year long file in minutes. PKH0034, Patient, Female, 61, Jordan
	49	Then next month when you return, you meet another doctor…It is a very big problem because the only person who knows my information, if I met with Dr X. S/he knows my information. PAT04-001, Patient, Male, 54, South Africa
	50	If the adjustment is done by a doctor with whom we get treatment regularly, this alone gives me more confidence because they know my medical record. 1007, Patient, Male, 60, Thailand
	51	(Seeing a different doctor] is not that big of a deal. Everything is typed down on the computer system. PKH0026, Patient, Female, 49, Jordan
Structures enabling flexibility in service delivery and care practice.	52	I think the routine check-up should be done at a narrower interval, it should be tailored to each individual case. CKH0008, Caregiver, Female, 31, Jordan
	53	Then we have got the stable patients…and for them we expect them to fit into quite neat boxes of attending on certain days and collecting medication, which might not fit exactly with how often they feel they need to be seen but that is how it is in a big, clumsy system. PROF07-002, HCP, Female, 49, South Africa
	54	If the patient’s condition is well, the doctor would schedule each appointment far apart. If it is not well, the doctor would schedule frequent appointments. If there is a problem, I can come before the appointment. This kind of appointment system is good because it is flexible and made according to the symptoms. 1013, Patient, Male, 50, Thailand
Establishing cooperation pathways across specialisms and institutions	55	If we had better coordination with other departments, this would make things much better for the patients and would reduce our load. HCP19, HCP, Male, 55, Jordan
	56	It would be great if there were a way to enhance a communication between staff from different hospitals such as using social network platforms which would be better that contacting by phone. It will help patient to feel more confident to visit a hospital near their home if they know that we have a connection with a local hospital and we can share patients’ information between hospitals. 3001, HCP, Female, 26, Thailand
Family and friends’ involvement and support	57	In Syria, I would be surrounded by my family and relatives, but here I have no one. CKH007, Caregiver, Male, 54, Jordan
	58	My family are caring and they keep checking on me, whether it was my family or my in-laws, they support me emotionally as much as they can. PAL0040, Patient, Female, 43, Jordan
	59	We are a prayer group but they are always there for me. They were all over 80. They are mothers and fathers to me…they are always there for me. They are always there, when I am down, they come and they give me home meditations and when I am in hospital, then they come to hospital. PAT01-002, Patient, Male, 58, South Africa
	60	Well-to-do patients are taken care of well by their families. Certain families hire caregivers to look after the patients so they don’t have to take leave from work to do the care by themselves. However, needy patients usually go to hospital by themselves because they have no relatives looking after them. 3012, HCP, Female, 20, Thailand
Involving family/friends in information-sharing and decision-making	61	I believe skills should be passed on to all people surrounding the patient because when a person gets sick, all of those around them are also sick…When you see a person going through so much pain and you can’t do anything about it, that makes you feel guilty… it is nice to provide guidance. PKH0017, Patient, Male, 49, Jordan
	62	Sometimes he is like that, like when he had the pain on his chest or, and then I do not know what to do. I would like to know what I can do in that time when he is like that. CAR03-007, Caregiver, Female, 38, South Africa
	63	Also the families to also be involved in the health education so that they can try to assist the patient, because when the patient becomes short of breath they tend to forget the techniques of how to use the pumps and they do not use it the right way and it does not get effective and they panic. PROF08-001, HCP, Male, 52, South Africa
		Information should also be given to relatives so that they can help me get better. My relatives will remind me to follow the doctor’s advice because I can forget sometimes. 1008, Patient, Male, 63, Thailand
	64	(My sister) has a hard life, she sleeps at my place and leaves her little son at home, she worries about the radiation from chemo affecting him, she is on the phone all night long while he is crying. PKH0049, Patient, Female, 56, Jordan
	65	I stop my studies so that I could take care of her. CAR04-002, Caregiver, Female, 26, South Africa
Addressing the needs of family/friends	66	Since the patient got sick, I haven’t sold any goods; it’s been two years now. Her illness has greatly affected me as I’m stressed and sleepless. 2010, Caregiver, Female, 52, Thailand
	67	Our life is full of sadness and depression. CKH009, Caregiver, Female, 44, Jordan
	68	You also need to put at ease the spouse – the family member. Or sometimes it’s the children of patients…And for them, when they see someone battling with breathing…what comes into their minds is death. So, you need to stabilise psychologically. PROF03-002, HCP, Female, 28, South Africa
	69	Her illness has greatly affected me as I’m stressed and sleepless; I have to take a sleeping pill every night. 2010, Caregiver, Female, 52, Thailand
	70	Mental care should be provided [to family members] by the entire hospital, not just this clinic. There should be someone to listen to and encourage the family members. We should provide bereavement care. 3011, HCP, Female, 25, Thailand
	71	
Promoting continuation of normality and self-identity	72	Mentally, I don’t feel good enough every morning to go out to work, sometimes my body would be good but I wouldn’t feel like it, but mostly the reason is physical…I heard that the hospital used to get jobs for patients like me, I wish they could do that. PKH0027, Patient, Male, 28, Jordan
Support for participating in regular personal life activities	73	I want my pain to calm down so that I can be there for (my children). Even if I still have the disease. I want to wake up in the morning, make coffee, I don’t have to cook, I can fix myself something simple, move around the house. I want to reduce the pain so I can be around them again. PKH0011, Patient, Female, 44, Jordan
	74	I slowed down socially. Like we used to like going to dance and I found that I cannot be on the floor long late of lately. PAT01-004, Patient, Female, 74, South Africa
	75	She just completed her high vocational course but hasn't received the certificate. After graduation, she was then hospitalised. She wants to pursue her study for two more years; now that she falls ill, she complains about it every day that she wants to study. This makes her stressed, sad, and sleepless. 2010, Caregiver, Female, 52, Thailand
Providing meaningful activities for inpatients	76	I want some fresh air…we haven’t left this place since last Monday, I feel like I have forgotten how the outside world looks like…I want my sister to see the world and see other people. CKH0020, Caregiver, Female, 45, Jordan
	77	He got stressed and tried to elope from hospital. He felt very bored. 2005, Caregiver, Female, 50, Thailand

HCP, healthcare professional.

#### Processes

The data also aligned with the two process domains of PCC derived by systematic review^7^ : *Family and friend involvement and support* and *Promoting continuation of normality and self-identity*. Patient experiences showed wide variation in the level of support received from family members and friends (quotes 57–60). It was suggested that HCPs must carefully consider whether or not a patient has a social support network, and which family members or friends, if any, can be drawn on to help support the patient (quotes 61–64). Proactively providing family and friend caregivers with information about the person’s diagnosis, symptoms and side effects was deemed critical in enabling caregivers to confidently care for the person and avoid feelings of helplessness (quotes 61–62). Sharing information with caregivers was also considered beneficial as they could sometimes better understand or remember information and self-management techniques and could later remind or assist the patient with these (quotes 63–64). The data also highlighted the importance of *addressing the needs of family members and close friends.* Serious illness was described as having a wide variety of impacts on the lives of caregivers including taking time away from work or education to care for the patient; financial challenges resulting from healthcare costs or reduced ability to work; less time spent with their children or other family members (quotes 65–67). Participants also described the impacts of a person’s serious illness on the psychological health of their loved ones and believed that psychological support should be provided for family members during the course of a patient’s diagnosis, care, treatment and death (quotes 68–71).

*Promoting continuation of normality and self-identity* was also raised by patients, caregivers and HCPs. Interviewees reported the significant impact of serious illness on a person’s ability to carry out their normal roles and daily tasks and their future plans and life aspirations. This in turn impacted their confidence, sense of self and identity (quotes 72–75) Patients and caregivers suggested the need to *support patients with participating in regular personal life activities,* including socialising, employment and religious activities. Reportedly, this support should aim to control symptoms most interfering with a person’s daily tasks, advise individuals on strategies to adapt their life to live with their disease and redirect their focus to the activities and people that matter most to them (quotes 72–77).

### Inductive overarching themes (each with structures, processes and outcomes)

Five additional overarching themes were identified from the data through inductive reasoning: interdependency and collectivism; bringing care into the home and community; equity and non-discrimination; understanding and addressing health within the context of limited resources and workforce well-being. Table 4 displays illustrative quotations for these themes.

**Table 4:** Illustrative participant quotations for inductive PCC themes (for more in-depth table of inductive themes and illustrative quotations see online supplemental table 3)

Inductive theme		Illustrative quotations
**Collectivism**		
Benefits of a support system and positive relationships on well-being The importance of relationships Interdependence of well-being Value of condition-specific patient and family support groups	78	I have a group of friends who would pull me out of the hole every time I felt depressed and they keep telling me what a fighter…I am. Support groups are very important for cancer patients. PKH0017, Patient, Male, 49, Jordan
	79	These patients really need quite a lot of support because they are so dependent on those who are around them and what is happening around them. PROF07-004, HCP, Male, 50, South Africa
	80	I always come here with my family so I don’t feel stressed. My daughter can manage everything for me…where to contact and where to go. 1006, Patient, Female, 58, Thailand
	81	Interviewer: Are you afraid of death? Patient: No, I am afraid I might leave my children). PKH0030, Patient, Female, 33, Jordan
	82	I think I have grown too fast, maybe 20 years older than I should be, I am responsible for everything in the house, my father feels like a son to me, I would be measuring his temperature or blood pressure. CAL0005, Caregiver, Female, 27, Jordan
	83	The interesting thing is their families are quite frustrated with them. And I think they get a lifetime of dad being rude, irritable, maybe contributing to often quiet, sad broken homes. PROF04-001, HCP, Female, 39, South Africa
	84	It would be nice if we could help each other. For us too to help. To have something that we do in order to show other people that even us, we can do this, we just need to persevere. Yes, for us to persevere and not lose hope. PAT04-002, Patient, Male, 38, South Africa
	85	Their decisions depend on their relatives, not them. Patients will choose to do things that would not be a burden to their relatives. For example, patients who have to come to hospital often don’t want to come because they don’t want their relatives to take leave from work. 3007, HCP, Female, 27, Thailand
**Bringing services into the local community or home**		
Patient not wanting to be admitted/spend long periods in hospital Drawing on other human resources in community for example, health promoters or volunteers; traditional health workers; support from community or religious organisations Creating a health-promoting home environment	86	I would tell him I don’t want to be admitted…because I had recently moved to a new place and I want to stay with my children, and I would ask him if I can just take the chemo and go home. PKH0030, Patient, Female, 33, Jordan
	87	We start preparing the patient from the day of admission to the discharge. We get the equipment and with donations from the social worker, we get the proper mattress and generator and whatever else needs to be ready at their homes. HCP8, HCP, Male, 30, Jordan
	88	If we can get a multi-pronged approach, where we have social workers, homecare-based carers, we have home visits, we have social workers able to actually go to the communities and see what is available in the community and upskill those people, that will be very useful. PROF01-002, HCP, Male, 45, South Africa
	89	I think if they say we are struggling at home, we have the home-based care systems that the sisters come around but it is unfortunately also not an everyday support system. PROF01-008, HCP, Female, 26, South Africa
	90	Home visits will help us better follow up with patients’ symptoms. Relatives of bedridden patients will have trouble bringing the patients to the hospital. If we can pay a home visit, it would be convenient for them. 3009, HCP, Female, 21, Thailand
	91	A village volunteer’s visit would be nice to help patients. If the patient’s condition worsens, there would be someone to inform the relatives to take the patient to the hospital. 1010, Patient, Female, 60, Thailand
**Equity and non-discrimination**		
Wish to be treated with equal respect and care irrespective of socioeconomic status Ensure services are accessible and understandable by persons of all socioeconomic backgrounds, nationalities and ages Underprivileged patients and families in need of additional social support Patients and carers wish for most urgent cases to be prioritised Impacts of pre-existing relationship between patient and HCP Carefulness required when communicating with people of diverse ethnicities/nationalities Sex and gender impacts a) communication between HCPs and patients or family members b) impacts of illness on a person’s life	92	*They don’t have to stick to the line, they should let in patients who have a more serious condition that others. PKH0016, Patient, Female, 63, Jordan*
	93	Sometimes I think it is how much you earn and how much money you get in your bank account…I think if you have lots of money, people will help you with respect. If you got less, they treat you like nothing sometimes. CAR03-008, Caregiver, Female, 39, South Africa
	94	I think more emphasis in identifying patients who need more help. PROF01-001, HCP, Female, 30, South Africa
	95	I am at an advantage because I know someone who knows someone…when I come to the clinic it’s about who I know. That will determine whether or not I will be seen or assisted. CAR04-002, Caregiver, Female, 26, South Africa
	96	If the doctor talks about other things with the patients, it will slow down the patient’s examination. Other patients are waiting, so I have to be considerate. 1010, Patient, Female, 60, Thailand
	97	The law here states that foreigners are charged more than citizens, so it is not discrimination, it is just the law. CAL0017, Caregiver, Male, 41, Jordan
	98	(Patients) have different education and socioeconomic backgrounds. They should have some knowledge so that they can understand this kind of service system. I think that doctors, nowadays, have improved in their communication skills so they can talk to patients. 1005, Patient, Male, 52, Thailand
	99	I’m not good at communicating via the Internet like this clinic does as I'm old-school and not good with computers. 1007, Patient, Male, 60, Thailand
	100	There’s a lot of socialization during the visits. You’re discussing things with someone from a different culture and you both have your questions. HCP06, HCP, Female, 31, Jordan
	101	I like the Afrikaans doctors because they understand me better. [I can speak] my language. So, I cannot say that the foreign doctors are incapable of doing that. They are capable of doing their job but sometimes you just feel like they do not examine you nicely because you do not tell them. PAT03-001, Patient, Male, 42, South Africa
	102	I come from a masculine family, as all of my 7 brothers are older than me. They had all the power and us the girls, we had no opinion. PKH0012, Patient, Female, 50, Jorda
	103	Another thing that upsets me is that I don’t get my period anymore, so it feels like my life is over…I really like kids but now even if I decided to get married, I won’t be able to have kids. Nobody is gonna want a woman with cancer, let’s be realistic. PKH0020, Patient, Female, 42, Jordan
**Context of limited resources**		
Limited financial and human resources for health services impacts ability to provide PCC Limited personal resources of patients and families—poor housing and employment conditions contributing to poor health and well-being Patients using health financial assistance or selling therapeutic equipment to cover basic living expenses Material realities impose constraints on patient ability to engage or adhere Adjusted preferences and expectations	104	I end up taking it upon myself to provide these services including coordination, palliation, radiotherapy, chemotherapy, and spiritual support as well. I end up feeling unsatisfied with myself thinking that I haven’t done enough, when in reality I can’t. HCP13, HCP, Male, 35, Jordan
	105	There would be some policy, financial, personnel, and equipment issues (to provide better care). Not all hospitals have the necessary resources. 2009, Caregiver, Female, 45, Thailand
	106	I stopped the treatment for financial reasons. PAL0006, Patient, Male, 68, Jordan
	107	Our housing system is not alright, some of these patients are coming from informal settlements. So, it’s crowded…so even if moss I don’t smoke, if next door there is smoking. PROF03-002, HCP, Female, 28, South Africa
	108	Some of the patients sell their pumps to get money and then they come back to the facility, tight chest and we need to give them another pump.” PROF07-003, HCP, Female, 55, South Africa
	109	People are going to say you have to eat healthy and then a lot of people are going to say but how are we going to eat healthy because it is reality you have to eat what you have…nowadays living healthy is very expensive. CAR03-011, Caregiver, Female, 42, South Africa
	110	We should know about personal information that might affect patients’ condition such as their jobs because certain jobs can trigger a relapse. 3009, HCP, Female, 21, Thailand
	111	Some patients have to take medicines that are not covered by the Universal Health Coverage Scheme. Some [ask] us whether they could stop taking that medicine…because it was so expensive. 3006, HCP, Male, 24, Thailand
	112	Patient’s satisfaction rate is excellent because as I told you we serve a category of people that are mostly livening a simple lifestyle, they don’t require luxurious treatment, they need core simple services that can simply satisfy them and make them happy, such as, doctors, nurses, pharmacy and scans. HCP16, HCP, Male, 35, Jordan
	113	You have to answer questions from scratch about medical history which can get annoying, but then you are not going to be annoyed because now you are getting a free service. CAR04-002, Caregiver, Female, 26, South Africa
**Healthcare workforce well-being**		
Overstretched and under-staffed Criticism or verbal abuse from patients Psychological challenges of working with end-of-life patients	114	It’s also exhausting to see 15 palliative care patients on the same day. You need to rest in between patients and recharge so you can provide the best service. HCP12, HCP, Female, 28, Jordan
	115	Sometimes we give more than we take. My salary is 320 JD and I get 100 JD incentives every 3 months. I worked 100 times worth that salary for 10 years. CKH0009, Caregiver (and nurse), Female, 44, Jordan
	116	I would like to hear about the experiences of my colleagues in the same field. Did they experience the same things I did? Are my feelings normal?…Emotional support is the most important thing we as healthcare providers require. HCP4, HCP, Female, 31, Jordan
	117	I just feel that a person brought his/her problem from the house…Now s/he is out of control. There is exhaustion. The pressure is high. CAR04-001 Caregiver, Female, 48, South Africa
	118	Even if the psychological support existed in a more formal in-depth way, I am not sure how well it would be utilised because just there is work to be done and sometimes I will just carry on because I would rather get home at a normal home going time. PROF01-005, HCP, Male, 32, South Africa
	119	I used to give patients my personal Line contact in case they needed medical advice. It turned out to be too much for me. They were not considerate. When I could not help, I got criticism. ID3007, HCP, Female, 27, Thailand
	120	I think some staff may need to see a psychiatrist in order to vent their feelings so that they can smile when they see patients…and see…how much they help other people. ID 2005, Caregiver, Female, 50, Thailand

HCP, healthcare professional; PCC, person-centred care.

#### Interdependency and collectivism

An overarching theme within the data was ‘collectivism’: the tendency, on the individual and societal level, to view oneself as interdependent and a member of a group, rather than as an atomistic independent being. Participants across all countries and conditions discussed the benefits of social support systems (quotes 78–80), the necessity of social interactions for human well-being (quote 78), the value of interpersonal harmony, the impacts of serious illness on close relationships and familial roles (quotes 82, 83) and the interdependence of a person’s and their loved ones’ well-being (quotes 81, 85). Participants also pointed to the value of group-based learning and peer-to-peer support (quote 84).

#### Bringing care into the home and community

Patients and caregivers commonly expressed a preference for spending minimal time within healthcare facilities, instead wanting to return to their home and families (quote 86). Participants across all countries stressed the value of home visits, equipping the home for their needs, setting up a health-promoting home environment and upskilling the patient and patient’s household in self-management (quotes 87–91). Reducing patients’ need to travel to facilities was further seen to prevent exhausting unwell individuals and improve care access for those living far away from facilities (quote 90). To provide care in the home and community, participants suggested drawing on, upskilling and coordinating with human resources from the community, including health promoters, traditional health workers, volunteers and local social workers (quotes 88, 91).

#### Equity and non-discrimination

Patients and caregivers displayed consideration for one another and generally desired for all patients and caregivers to be treated with equal care and respect by HCPs irrespective of their financial status or nationality, and for cases to be prioritised solely on the basis of need (quotes 92–96). A widespread view was the need to ensure services are accessible to and understandable by persons of all socioeconomic backgrounds (quotes 97–99). Participant’s across all countries also described the additional care required when communicating with people of diverse ethnicities and nationalities and suggested the need to ensure that such diversity is considered and accommodated in care provision (quote 100, 101). For participants based in Jordan, while it was believed that patients did not face any discrimination from HCPs based on their nationality, discriminatory healthcare access and financing were described, with non-nationals often experiencing significant financial burdens and barriers to accessing healthcare (quote 97).

Participant experiences and views also revealed the multifaceted impacts of sex and gender roles on clinical interactions and communication, and on the life consequences of serious illness. For example, in the Jordanian data, it was often reported that female patients and caregivers were left out of conversations with HCPs and decision-making despite their preference for involvement (quote 102). These patients also commonly described the sex-specific impacts of serious illness on their lives, including an inability to have children and reduced prospects of marriage (quote 103).

#### Understanding and addressing health within the context of limited resources

Participants across all countries and stakeholder groups described a context of limited financial and human resources for health facilities, including staff shortages and unavailability of appropriate medications, equipment and facilities; HCPs often saw this as an obstacle to providing care that was responsive to persons’ needs (quotes 104, 105).

Participants across all three countries also discussed the limited personal resources of many patients and families, and the ways in which disadvantaged social conditions, including poor housing, employment, education and financial status, contributed towards making a person unwell (quotes 106–111). Participants described the constraints that material realities imposed on a person’s ability to adhere to treatment or advice. For example, participants described inability to consume a healthy diet because of financial constraints (quote 109), an inability to continue with a treatment plan due to high costs (quotes 106, 111) and patients selling their own medical equipment to combat poverty (quote 108).

Low personal resources and disadvantaged social circumstances also appeared to shape patient and family priorities. Patients and caregivers appeared to show adjusted preferences and expectations, either lowering their expectations in line with available resources or showing a low sense of entitlement to higher quality care (quotes 112, 113).

#### Workforce well-being

Participants from all stakeholder groups and all countries often reported health facility working conditions that negatively affected HCPs’ well-being and capability to care for patients. A widespread view was that healthcare staff were overstretched and overworked on a day-to-day basis (quotes 114, 117). Additionally, HCPs sometimes faced strong criticism or verbal abuse from patients, and some HCPs felt that they were inadequately compensated for their work (quotes 115, 119). Participants also reported the psychological challenges of caring for seriously unwell patients and suggested the need for psychological and emotional support for HCPs and opportunities to debrief with colleagues (quote 116, 120). However, a few HCPs questioned the feasibility of engaging in psychological support, expressing concern that such activities would take away from clinical time with patients and further increase their time spent at work (quote 118).

## Discussion

The findings both reveal specific practical actions that contribute towards delivering PCC and identify new cross-national domains of person-centredness. In focusing on LMIC settings and serious conditions, the data challenge particular assumptions and clarify ambiguities in existing PCC models and identify new areas of focus.

Firstly, the results indicate that certain commonly advocated PCC principles may be viewed differently by persons of more collectivist cultural backgrounds. Some individuals may place more value on support practices that facilitate family and community activities rather than individual autonomy and competence. As discussed by Setlhare *et al*^8^ and Markus and Kitayama,^29^ while Western notions of the ‘self’ are that of an individual independent agent, in most non-Western societies the ‘individual’ is more integrated with significant others, largely finding identity and value through her community and aligning individual interests with community preferences. PCC theory must accommodate this worldview, move away from solely individualistic conceptualisations and promote activities that enable people to support each other, for example, establishing condition-specific patient and family support groups and cultivating positive communication and relationships between a patient and their family, friends and colleagues. This supports conceptualisations of ‘people-centred’ care, such as that by the WHO, which brings attention to the health of people within their full social circles and communities.^30^,^32^ In line with this collectivist view, the study findings also suggest the need for community-based and home-based care. This may be enhanced by providing home visits to advise patients and families on symptom control and health behaviours, and by building links with existing human resources in the community. Attention and resources must be directed towards community-based health workers and outreach activities, rather than skewed towards facility-based care. This aligns with WHO estimates that 70%–90% of all healthcare takes place in the home.^33^

In viewing patient engagement from a more collectivist perspective, our PCC model better highlights broad considerations of social circumstances. The study findings indicate that PCC must consider and go some way to address the social determinants of health and disease, healthcare access and health inequities, including housing conditions, education opportunities, employment and societal gender roles. HCPs must be supported to assess and address how social realities may be influencing a person’s health and affecting their ability to engage with, or adhere to, advice or treatment plans. Where possible, person-centred health interventions must be tailored to take into account prevalent negative social determinants. This supports work by Maeseneer *et al*,^31^ who suggest that *people-*centred care must give due attention to the social determinants of health and bridge the gap between individual treatment of disease and community-oriented public health actions. This finding also aligns with May *et al*’s view that delegating the ‘work’ of managing illness to patients and families risks overwhelming them and worsening outcomes if they do not have the resources to achieve these ends.^34^

The data also indicate diverse personal preferences regarding particular components of PCC, for example, level of patient involvement in decision-making. This requires us to differentiate between a person making an informed *choice* not to perform a particular function, versus a person being *unable* to realise a particular capability due to personal or societal circumstances. We, therefore, support that PCC should aim to cultivate particular capabilities,^35^,^37^ giving a person the genuine opportunity to do and be the things that matter to them, including making informed decisions about treatment plans through developing their health literacy, spending quality time with loved ones, performing daily tasks, etc. To achieve this, HCPs should consider which set of capabilities a patient or family member already has, which capabilities they value, and then strive to develop valued underdeveloped capabilities, taking into consideration person’s situational barriers. We suggest that these activities should be measured and monitored as process outcomes. Conceptualising PCC in terms of capabilities in this way takes account of personal freedom and avoids imposing particular non-universal ideals on all persons, while recognising the diverse social influences on what a person is able to do and be.

The study findings further highlight that PCC requires and should enable well-being of the workforce. Burnt out and depersonalised practitioners are both less able to offer PCC to patients and families and are an indicator of non-PCC in itself. This aligns with previous calls for PCC to recognise the personhood of HCPs, both for the intrinsic and instrumental value.^35 38 39^ Psychological support should be provided for HCPs working with seriously ill patients, and time allocated for rest and opportunities to debrief and share experiences with colleagues. Structural, organisational barriers to delivering PCC must be assessed and tackled to avoid feelings of helplessness and high stress among HCPs.

Importantly, the results of this study indicate that PCC must centre around a shared set of human values, including equity, non-discrimination, compassion and non-judgementalism from HCPs. PCC should promote equal opportunity to be healthy for all persons and ensuring that diversity in race, ethnicity, religion, sex, age, sexual orientation and socioeconomic status is considered and incorporated. Such values must be promoted in workforce recruitment, training and performance assessment and enacted through skilled communication and decision-making. This conclusion aligns with existing work on values-based practice^40^ and with Rider *et al*’s International Charter for Human Values in Healthcare,^41^ which stresses that human dimensions of healthcare are fundamental to compassionate, ethical and safe care.

Finally, the findings indicate that PCC outcomes that are important to measure include quality of life, ability to engage in daily life activities, progress towards personal goals and valued capabilities and well-being of a patient’s loved ones, rather than merely disease progression or health status improvement. Furthermore, the adjusted preferences seen among participants imply that caution must be taken when collecting or interpreting patient-reported experience data; seemingly high satisfaction may be influenced by low expectations or low sense of entitlement and may sometimes conceal important limitations of the care provided.^42^

### Strengths and limitations

To our knowledge, this study is the first cross-national, cross-condition exploration of the meaning and acceptability of PCC in LMIC settings. In focusing on three highly diverse countries and three serious conditions, a diversity of experiences, values and expectations have been captured, enabling a theory with greater depth and applicability. Collaboration across interdisciplinary, multinational researchers also allowed a range of perspectives and cultural viewpoints to inform the data collection, analysis and interpretation. Further depth and applicability of the findings may have been achieved by inclusion of study sites in low-income countries and in rural settings. We recognise that the practice of PCC is likely to involve further challenges in resource-constrained settings.^43^ Future research could also be conducted with healthcare manager or policymaker participants to investigate the structural PCC domains proposed by Santana *et al* that were not discussed by participants in this study yet may be important.

A study limitation is the lower sample size from the Thai study site relative to other country sites. This is due to data collection at this site partially coinciding with a local rise in COVID-19 cases. Data collection processes were altered in response, including recruitment during routine telephone appointments and giving potential participants the option to participate via telephone. Nevertheless, recruitment was slowed considerably, and the researchers were forced to stop data collection before reaching the anticipated sample size. Despite this, the high quality of the data collected in Thailand generated sufficient information power^25^ for meaningful conclusions to be drawn.

## Conclusion

The study findings both indicate specific practical actions that can contribute towards delivering PCC and highlight new cross-national domains of person-centredness. The data suggest that PCC requires particular structural features of the healthcare system to be in place, such as professional education in person-centred values, opportunities for HCPs to share experiences and partnerships with community-based workers. These structures better enable important PCC processes to be enacted, including tailored, compassionate communication, information sharing and engaging patients and their families in care decisions. These processes must provide genuine opportunities for patients and families to do and be the things that matter to them, such as making informed decisions about their care, performing daily tasks and engaging in social relationships. PCC structures, processes and outcomes must accommodate a collectivist perspective, support the well-being of the workforce and promote a common set of human values.

## Supplementary material

10.1136/bmjgh-2022-008843 online supplemental file 1

10.1136/bmjgh-2022-008843 online supplemental file 2

10.1136/bmjgh-2022-008843 online supplemental file 3

10.1136/bmjgh-2022-008843 online supplemental file 4

10.1136/bmjgh-2022-008843 online supplemental file 5

10.1136/bmjgh-2022-008843 online supplemental file 6

10.1136/bmjgh-2022-008843 online supplemental file 7

10.1136/bmjgh-2022-008843 online supplemental file 8

## Data Availability

All data relevant to the study are included in the article or uploaded as supplementary information.
