# Supplementary material for: Developing a global practice-based framework of person-centred care from primary data: a cross-national qualitative study with patients, caregivers and healthcare professionals
Source: BMJ Glob Health. 2022 Jul 13;7(7):e008843. doi: 10.1136/bmjgh-2022-008843 (PMC9280875; doi:10.1136/bmjgh-2022-008843)
Supplement: online supplemental file 1 [file bmjgh-2022-008843supp001.pdf]

Appendix A. Summary of Country Characteristics

	Jordan	South Africa	Thailand
WHO region [1]	Eastern Mediterranean	African	South-East Asia
Most spoken first languages [2, 3]	Jordanian Arabic (60%)	Zulu (23%)<br>Xhosa (16%)<br>Afrikaans (14%)	Central Thai (29%)<br>Lao (22%)
Predominant religion [4]	Sunni Islam	Christianity	Theravada Buddhism
Population [5, 6]	10 million	57 million	69 million
Population distribution [7, 8]	85% urban	65% urban	52% urban
Population living below national poverty line [8-10]	17%	55%	10%
World Bank country economy classification [11]	Upper-middle-income	Upper-middle-income	Upper-middle-income
Healthcare provision landscape [12-14]	Public; Private; strong humanitarian agency presence	Public; private	Civil service welfare system for civil servants; social Security for private employees; universal coverage scheme for other citizens

Table references

1. https://www.who.int/countries.
2. https://www.ethnologue.com/country/JO/languages.
3. *International Convention on the Elimination of All Forms of Racial Discrimination; Reports submitted by States parties under article 9 of the Convention: Thailand (PDF). United Nations Committee on the Elimination of Racial Discrimination.* . 2011.
4. *Religious Affiliation Pew Research Center.* , in *The World's Muslims: Unity and Diversity*. 2012, Pew Research Center.
5. Division, U.N.P., *World Population Prospects: 2019 Revision.* 2019.
6. *Mid - year population estimates" (PDF). Stats SA. Statistics South Africa.* 23 July 2018.
7. https://tradingeconomics.com/thailand/rural-population-percent-of-total-population-wb-data.html. [cited 2019 13th September].
8. https://tradingeconomics.com/south-africa/rural-population-percent-of-total-population-wb-data.html. [cited 2019 13th September].
9. *"World Databank". Databank.worldbank.org. 2015.* . [cited 2019 13th September].
10. *The World Bank. Country data.*
11. https://datahelpdesk.worldbank.org/knowledgebase/articles/906519-world-bank-country-and-lending-groups
12. Nazer, L.H. and H. Tuffaha, *Health Care and Pharmacy Practice in Jordan.* The Canadian journal of hospital pharmacy, 2017. **70**(2): p. 150-155.
13. Ataguba, J.E.-O., *Health Care Financing in South Africa: moving toward universal coverage* Continuing Medical Education, 2010. **28**(2).
14. Viroj Tangcharoensathien, W.W., Warisa Panichkriangkrai, Walaiporn Patcharanarumol, Anne Mills, *Health systems development in Thailand: a solid platform for successful implementation of universal health coverage.* The Lancet 2018. **391**.
