# Supplementary material for: Developing a global practice-based framework of person-centred care from primary data: a cross-national qualitative study with patients, caregivers and healthcare professionals
Source: BMJ Glob Health. 2022 Jul 13;7(7):e008843. doi: 10.1136/bmjgh-2022-008843 (PMC9280875; doi:10.1136/bmjgh-2022-008843)
Supplement: online supplemental file 2 [file bmjgh-2022-008843supp002.pdf]

Appendix B. Topic Guides

TOPIC GUIDE: IN-DEPTH INTERVIEWS FOR PATIENTS

Opening statements

Thank you for agreeing to take part in this research interview. My name is ________ and I am a member of the team working on this study. Today we will have a conversation about your illness, how it affects you, about your experiences of care, and how you would prefer to have your care provided. I am interested in hearing your thoughts so please do feel free to openly tell me what you think. The interview should take about 40 minutes. I will record our interview, but I will not start recording until we are ready to talk and I will tell you when I am switching it on.

We may cover some sensitive topics during the interview. You can pause or stop the interview at any point or skip any questions should you wish to. If anything I ask isn't clear, or you would like time to think about your answer, or you would like to raise something that you think is important, then please do tell me. I would like this to be a conversation so that I can find out what you think and what matters to you as someone living with heart failure.

What we talk about will not be shared directly and I will take out any names or places so that when I describe what patients have told me, they will not be able to know who I have interviewed. Also I will not share anything directly about you with your care team. However, if I am worried about your safety or anyone else's safety then I will let your care team know and I will let you know that I am doing that.

Could I please just confirm that you have signed consent form and read the information sheet? And are you happy for the interview to be audio recorded today? Do you have any questions before we start? Ok, I am turning on the recorder –

(START RECORDING)

1. Could you tell me a bit about yourself?
   Would you mind telling me a little bit about when you first became unwell?

2. Please could you tell me about your current experience of living with your disease – On a day-to-day basis, what are your main needs, priorities and concerns ?

a) How does your illness affect you on a day to day basis? What are your **physical and psychological needs?**

b) What have been the effects on your life **socially?**

c) What are your **religious or spiritual** needs, if any?

d) What would you say are your **main priorities** at the moment? Are there certain aspects of your life or your health that are most important to you at the moment?

e) Is anything in particular causing you **concern or distress**? What would you say are the things you'd most like to be managed? (e.g. symptoms, unmet needs)

f) How do you think your illness will affect you in the future? Do you have any particular concerns or worries about the future

3. [Referring back to any specific points raised above] - In what ways are [these needs] being met by the health services? In what ways are they not being met?
a) In what ways are your **needs** (physical/psychological/social/religious/spiritual) **and personal values being assessed and met**? In what ways are they not?<br><br>b) Could you tell me about whether the **people managing your care listen to you and understand your needs and concerns** and those of your **family/friends?**<br><br>c) Has your care been **tailored to your needs** in any way, and if so, how?

**4.1** How would you describe the **care that you have received** for your condition?<br><br>4.2 How would you **like** care to be delivered to you? (What would 'good' care look like to you? How do you feel that your care could be made better?)
a) How do you feel about your **physical caring environment** when you go to the community health clinic/district hospital? Do you feel safe?<br><br>How would you like the caring environment to be?<br><br>b) What kind of **support** did you receive? How did you feel about that support?<br><br>What could doctors/nurses do to better understand your needs and support you?<br><br>c) When you go to the community health clinic/district hospital, **how are you treated as an individual**? How do care staff behave towards you?<br><br>How would you like them to behave towards you? What things should doctors and nurses do to better understand you as a person?<br><br>d) At the community health clinic/district hospital, how have health professionals **communicated and interacted with you?** And **with your family/friends**? What have they asked you and your family/friends about? What problems do you think they expect you to talk about at your appointments? Are there any of your problems that you think they would not expect you to talk about?<br><br>How would you like your doctors and nurses to communicate with you and your family/ friends? What would you like them to ask you about and know about you? What could be done to make sure that the people that deliver your care interact with you [in the ways that you want]?<br><br>e) How have you found the **communication between the team** of people managing your care? How have they worked together? Have they collaborated/coordinated? Have they kept each other, and you, informed?<br><br>How would you like them to work together?<br><br>f) What do you think about the way in which any **information has been given to you about your disease and care**? How has it been provided? Is it enough information? Have you had the opportunity to ask questions? Have you felt able to ask the questions that are most important to you? If not, why? Do you get the answers and information that you want?

What are the most important questions for you about your disease? What would you like to know about your disease and care? How would you like that information to be shared? Is there anything you would **not** want to know about your disease and care?

g) What do you think about the way in which **information has been given to your family/friends** and the extent to which they have been involved?

How involved would you like them to be?

h) When there are any decisions or choices to be made about your care, who **is involved in that decision making**? To what degree are you involved in those decisions?

To what degree would you like to be involved in those decisions? How could we help you to feel part of those decisions? Who would you like to be involved in that decision making?

i) How would you describe the way in which your care has been **planned and coordinated**? Could you tell me about how your different appointments are scheduled? Where has your care been provided at different stages of your treatment? How often do you go to the community health clinic/district hospital? How do you decide when to go?

How would you like your care to be coordinated? E.g. How would you like your appointments to be arranged? Where would you like to be cared for?

j) Has anyone ever talked to you about how your care needs might change over time, **and about your future wishes and priorities for care**?

Would you want to have that discussion about your care in the future, and why? How would you like your future wishes and priorities for care to be discussed?

k) How **in control of managing your own health** do you feel? What do you do yourself to manage any health problems? What works best for you at home?

How would you feel about being more/less in control of managing your own health? How would you feel about you and your family being supported to care for yourself at home when possible? What would be good/bad about that and [If they respond positively] - How could we do that? What do you think could help you to manage problems at home?

5. Lastly I would like to ask you what you think might be the best possible way for us to give care to people when they come in to the hospital.

6. Would you make any changes or add anything to this interview?

Closing statements

If there is anything you would like to add about your experiences of care or anything we have missed out/not spoken about, you can always contact us afterwards. I'd like to thank you for taking the time to be interviewed today, we really appreciate it and your views will be a great help to us.

(END RECORDING)

General probes:

What does *[concept that arises/term they used e.g. 'respect', 'good appointment']* mean to you?
Could you tell me a bit more about how ____?
How does/would that happen?
How does/would that affect you?
How did/would you feel about that?

TOPIC GUIDE: PHASE 1 IN-DEPTH INTERVIEWS FOR CAREGIVERS

Opening statements

Thank you for agreeing to take part in this research interview. My name is _______ and I am a member of the team working on this study. Today we will have a conversation about your family member's/friend's illness, how it affects you, about your experiences of their care, and how you would prefer care to be provided. I am interested in hearing your thoughts so please do feel free to openly tell me what you think. The interview should take about 40 minutes. I will record our interview, but I will not start recording until we are ready to talk and I will tell you when I am switching it on.

We may cover some sensitive topics during the interview. You can pause or stop the interview at any point or skip any questions should you wish to. If anything I ask isn't clear, or you would like time to think about your answer, or you would like to raise something that you think is important, then please do tell me. I would like this to be a conversation so that I can find out what you think and what matters to you as someone caring for a family member/friend with heart failure.

What we talk about will not be shared directly and I will take out any names or places so that when I describe what family members/friends have told me, they will not be able to know who I have interviewed. Also I will not share anything directly about you with your care team. However, if I am worried about your safety or anyone else's safety then I will let your care team know and I will let you know that I am doing that.

Could I please just confirm that you have signed consent form and read the information sheet? And are you happy for the interview to be audio recorded today? Do you have any questions before we start?
Ok, I am turning on the recorder –

(START RECORDING)

2. Please could you tell me about your current experience of having a relative/friend with heart failure – On a day-to-day basis, what are your and your relative/friend's main needs, priorities and concerns?
a) How does your illness affect your relative/friend on a day to day basis? What are their **physical and psychological needs?**<br>b) What have been the effects on both your lives **socially?**<br>c) What are your and your relative/friend's **religious or spiritual** needs, if any?<br>d) What would you say are your **main priorities** at the moment? Are there certain aspects of your or your relative/friend's health that are most important to you at the moment?<br>e) Is anything in particular causing you **concern or distress**? What would you say are the things you'd most like to be managed? (e.g. symptoms, unmet needs)<br>f) How do you think their illness will affect you both in **the future**? Do you have any particular concerns or worries about the future?

3. [Referring back to any specific points raised above] - In what ways are [these needs] being met by the health services? In what ways are they not being met?

a) In what ways are your and your relative/friend’s **needs** (physical/psychological/ social/religious/spiritual) **and personal values being assessed and met**? In what ways are they not?

b) Could you tell me about whether the **people managing your relative/friend’s care listen to you and understand your needs and concerns** and those of your **relative/friend?**

c) Has your relative/friend’s care been **tailored to their/your needs** in any way, and if so, how?

4.1 How would you describe the **care that both your relative/friend and you have received**?

4.2 How would you **like** care to be delivered to your relative /friend? (What would ‘good’ care look like? How do you feel that your care could be made better?)

a) How do you feel about your relative/friend’s **physical caring environment** when you go to the community health clinic/district hospital? Do you both feel safe?

How would you like the caring environment to be?

b) What kind of **support** did you and your relative/friend receive? How did you feel about that support?

What could clinicians do to better understand your needs and support you both?

c) When you go to the community health clinic/district hospital, **how are you treated as an individual**? How do care staff behave towards you?

How would you like them to behave towards you?

d) At the community health clinic/district hospital, how have health professionals **communicated and interacted with you?** And **with your family member/friend**? What have they asked you and your relative/friend about? What problems do you think they expect your relative/friend to talk about at appointments? Are there any problems that you think they would not expect your relative/friend to talk about?

How would you like your doctors and nurses to communicate with you and your family/ friends? What would you like them to ask about? What could be done to make sure that the people that deliver your care interact with you and your relative/friend [in the ways that you want]?

e) How have you found the **communication between the team** of people managing your relative/friend’s care? How have they worked together? Have they collaborated/coordinated? Have they kept each other, and you, informed?

How would you like them to work together?

f) What do you think about the way in which any **information has been given to you and your relative/friend about their disease and care**? How has it been provided? Is it enough information? Have you had the opportunity to ask questions? Have you felt able to ask the questions that are most important to you? If not, why? Do you get the answers and information that you want?

What are the most important questions for you about their disease? What would you like to know about their disease and care? How would you like that information to be shared? Is there anything you would **not** want to know about their disease and care?

g) When there are any decisions or choices to be made about your relative/friend's care, who **was involved in that decision making**? To what degree was your relative/friend involved in those decisions? To what degree were you involved in those decisions?

To what degree would you like your relative/friend to be involved in those decisions? To what degree would you like to be involved in those decisions? How could we help you to feel part of those decisions?

h) How would you describe the way in which your relative/friend's care has been **planned and coordinated**? Could you tell me about how different appointments are scheduled? Where has their care been provided? How do they decide when to go to the community health clinic/district hospital?

How would you like their care to be coordinated? E.g. How would you like appointments to be arranged?

i) Has anyone ever talked to you and your friend/relative about how their care needs might change over time, **and about your future wishes and priorities for care**? How?

Would you want to have that discussion about their care in the future, and why? How would you like your and your friend/relative's future wishes and priorities for care to be discussed?

j) How **in control do you and your relative/friend feel in managing their health**? What do you both do yourself to manage their health problems? What works best for you at home?

How would you feel about them being more/less in control of managing their your own health? How would you feel about you and your family being supported to care for themselves at home when possible? What would be good/bad about that and [If they respond positively] - How could we do that? What do you think could help you both to manage problems at home and not go to the emergency department?

5. Lastly I would like to ask you what you think might be the best possible way for us to give care to people when they come in to the facility.

6. Would you make any changes or add anything to this interview?

Closing statements

If there is anything you would like to add about your experiences of care or anything we have missed out/not spoken about, you can always contact us afterwards. I'd like to thank you for taking the time to be interviewed today, we really appreciate it and your views will be a great help to us.

(END RECORDING)

General probes:

What does *[concept that arises/term they have used e.g. 'respect', 'dignity, 'good appointment']* mean to you?
Could you tell me a bit more about how ____?
How does/would that happen?
How does/would that affect you?
How did/would you feel about that?

TOPIC GUIDE: PHASE 1 IN-DEPTH INTERVIEWS FOR PROFESSIONALS

Opening statements

Thank you for agreeing to take part in this research interview. My name is _______ and I am a member of the team working on this study. Today we will have a conversation about your experiences of caring for people living with heart failure, how it affects them, your main challenges, and how you think we could improve the care provided. I am interested in hearing your thoughts so please do feel free to openly tell me what you think. The interview should take about 40 minutes. I will record our interview, but I will not start recording until we are ready to talk and I will tell you when I am switching it on.

You can pause or stop the interview at any point or skip any questions should you wish to. If anything I ask isn't clear, or you would like time to think about your answer, or you would like to raise something that you think is important, then please do tell me. I would like this to be a conversation so that I can find out what you think and what your experiences are as a healthcare professional caring for patient with heart failure.

What we talk about will not be shared directly and I will take out any names or places so that when I describe what professionals have told me, no one will be able to know who I have interviewed. Also I will not share anything directly about you or your patients with the rest of the care team. However, if I am worried about your, your patients', or anyone else's safety, then I will let other members of the care team know and I will let you know that I am doing that.

Could I please just confirm that you have signed consent form and read the information sheet? And are you happy for the interview to be audio recorded today? Do you have any questions before we start? Ok, I am turning on the recorder –

(START RECORDING)

1. Please could you tell me about your experiences of caring for heart failure patients – On a day-to-day basis, what are patient's and families' main needs, priorities and concerns?
g) How does the illness affect them on a day to day basis? What are their **physical and psychological needs?**<br>h) What are the effects on their life **socially?**<br>i) What about **religious or spiritual** needs, if any?<br>j) Of these problems and concerns, what would you say are the things are the most important to be managed?<br>k) How do patients/families talk about their illness affecting them in the future? Do they have any particular concerns or worries about the future?

2. [Referring back to any specific points raised above] - In what ways are [these needs] being met by the health services? In what ways are they not being met?

a) In what ways do you think patient’s **needs** (physical/psychological/social/religious/ spiritual) **and personal values are being assessed and met**? In what ways are they not? Are they well managed- if not why not, if yes then how?

b) What training and support would you need to manage these needs better?

c) Are you able to **tailor your patient’s care to their needs** in any way, and if so, how?

3.1 How would you describe the **care that patients receive** for their condition?
3.2 How do you feel that this care could be made better? What do you think ‘good’ care should look like?

a) What is the current **physical caring environment** like at the community health clinic/district hospital?

Do you think the caring environment to could be improved? How? How could these changes be made?

b) How **are patients understood and treated as individuals**? How do you feel towards your patients?

What things should clinicians ask to better understand patients as an individual?

What could we do to make sure that patients feel respected by the clinicians? What would make patients feel like they are treated with dignity?

c) How do you currently **communicate and interact with you patients?** And **with their family/friends**? What do you ask them and their family/friends about? What problems do you expect them to talk about at appointments? Are there any problems that you don’t expect them to talk about?

What is the most important thing that doctors and nurses should know about their patients to make care as good as can be? What do you think would be most helpful for clinicians to ask patients to help you really understand what matters to them? Do you think you know that already about your patients? Why? What are the important questions to ask that maybe we don’t ask at the moment? Why do we think we don’t ask them?

d) How easily do you feel able to collaborate and coordinate the care that you provide by working and communicating with you colleagues? Is it easy/difficult to keep each other informed? In what way? why?

Are there any ways in which you think you could be better coordinated as a team? How could you be supported to make that happen?

e) What information do you give to your patients **about their disease and care**? How do you provide that information? Do your patients ask questions?

Do you think patients and their families get the information that they want? Is there anything you would **not** want to tell them about their disease? Why? Is there any information that it is difficult for you to provide? How could that be made easier?

f) How much **information do you give to a patient’s family/friends** and to what extent are they involved?

How involved do you think they should be? How would it be possible to involve them more?

g) When there are any decisions or choices to be made about a patient's care, who **is involved in that decision making**? To what degree is the patient involved in those decisions? Why?

Why might it be good/bad to further involve them? How could we help patients to feel part of the decisions that are made about their care?

h) What's the **standard care and support** delivered for heart failure patients (who they see, how often, referrals pathways, communication with emergency/respiratory medicine)? How is this care **planned and coordinated**? What works well/not so well?

How do you think this could be made better? What do you think would help you better provide for patient's needs?

i) Do you/your colleagues talk to patients about prognosis and likely advancing of their disease in the future? Or about the risk of unexpected death during an exacerbation? Or how their care needs might change over time? Why?

What might you need to help you have these conversations?

j) How **in control of managing their own health are you patients**? What do some of your patients do themselves to manage any health problems?

What do you think about the idea of finding ways to help patients and families to better manage themselves at home? How could we help them to self-manage their health? What would be good/bad about that? How could we do that? What could we say or do to make patients feel more in control of their own health?

4. Lastly I would like to ask you what you think might be the best possible way for us to improve patient care.

5. Would you make any changes or add anything to this interview?

Closing statements

If there is anything you would like to add about your experiences or ideas for care or anything we have missed out/not spoken about, you can always contact us afterwards. I'd like to thank you for taking the time to be interviewed today, we really appreciate it and your views will be a great help to us.

(END RECORDING)

General probes:

What does *[concept that arises/term they have used e.g. 'respect', 'dignity, 'good appointment']* mean to you?
Could you tell me a bit more about how _____?
How does/would that happen?
How does/would that affect you?
How did/would you feel about that?
