# Supplementary material for: Developing a global practice-based framework of person-centred care from primary data: a cross-national qualitative study with patients, caregivers and healthcare professionals
Source: BMJ Glob Health. 2022 Jul 13;7(7):e008843. doi: 10.1136/bmjgh-2022-008843 (PMC9280875; doi:10.1136/bmjgh-2022-008843)
Supplement: online supplemental file 4 [file bmjgh-2022-008843supp004.pdf]

A priori coding frame

(Santana model codes are presented in black text; Giusti et al.'s systematic review codes are presented in blue text)

Structures

S1. Creating a PCC culture subdomain

S1a. Core values and philosophy of the organisation

- Vision, Mission
- Patient-directed: integrating patient experience and expertise
- Addressing and incorporating diversity in care, health promotion and patient engagement
- Patient and health-care provider rights

S1b. Establishing operational definition of PCC

- Consistent operational definitions
- Common language around PCC

S2. Co-designing the development and implementation of educational programs

Standardised PCC training in all healthcare professional programs

- Integration of all health-care sectors and professionals
- Professional education and accrediting bodies
- Translating into practice through continued professional education and mentorship
- Training in holistic perception of human organism
- Training for non-clinical staff in providing compassionate and co-ordinated PCC

S3. Co-designing the development and implementation of health promotion and prevention programs

S3a. Collaboration and empowerment of patients, communities and organisations in design of programs

- Identify resources
- Creating partnerships with community organizations
- Create patient advisory groups

S4. Supporting a workforce committed to PCC

S4a. Ensure resources for staff to practice PCC

- Identify resources
- Creating partnerships with community organizations
- Create patient advisory groups

S4b. Ensure strong team leadership around PCC

S5. Providing a supportive and accommodating PCC environment

S5a. Designing healthcare facilities and services promoting PCC

- Collaborate with and empower patients and staff in designing health-care facilities
- Environments that are welcoming, comfortable and respectful
- Spaces that provide privacy
- Spiritual and religious spaces
- Facility that prioritize the safety and security of its patients and staff
- Areas/rooms that will support the accommodation of patients

S5b. Integrating organisation-wide services promoting PCC

- Provide interpretation and language services
- Patient-directed visiting hours

S6. Developing and integrating structures to support health information technology

Common e-health platform for health information exchange across providers and patients

- Electronic Health Record systems with capacity to coordinate & share healthcare interactions across continuum of care
- Health information privacy and security
- E-health adoption support through strategic funding and education

S7. Creating structures to measure and monitor PCC performance

Co-design and develop framework for measurement, monitoring and evaluation

- Co-design and development of innovative programs to collect patients and caregiver experiences about care received and providing timely feedback to improve the quality of health care
- Reporting and feedback for accountability and to improve quality of health care

S.8 Structuring service organisation to enable continuity of care and patient navigation

S8a. Simplification of care pathways to ease patient navigation

S8b. Appointment system structured to allow patients to see same professionals over time

S8c. Structures enabling flexibility in service delivery & care practice

S8d. Establishing cooperation pathways across specialisms and institutions

Processes

P1. Cultivating communication

P1a. Listening to patients

- Gathering information through active listening
- Asking questions of what patients want to discuss (concerns, views, understanding)
- Non-verbal behaviours (eye-contact, listening attentively, proximity/touch, head nodding)

P1b. Sharing information

- Patients provided with all necessary information to make informed decisions relating to their diagnosis & treatment plan
- Sharing of information regarding patient's condition and their own impact/influences on their condition

P1c. Discussing care plans with patients

- Responding to patient and caregiver needs
- Aim and follow-up of treatment or interventions with possible outcomes and adverse events/side-effects
- Discussing and building capacity of patients for self-management and self-care
- Acknowledging and discussing uncertainties
- Creating a shared understanding

P2. Respectful and compassionate care

P2a. Being responsive to preferences, needs and values

- Acknowledge the patient as an expert in their own health and as a part of the health-care team
- Understanding patient within his/her unique psychosocial or cultural context (i.e: awareness of religious, spiritual, lifestyle, social and environmental factors)
- Responding empathically

P2b. Providing supportive care

- Acknowledge the patient as an expert in their own health and as a part of the health-care team
- Understanding patient within his/her unique psychosocial or cultural context
- Responding empathically

P2c. Promoting continuation of normality and self-identity

- Support for participating in regular personal life activities
- Providing meaningful activities for inpatients

P3. Engaging patients in managing their care

Co-designing care plans with patients

- Shared decision making
- Goal-setting
- Supporting self-care management
- Care plans can be accessed by patients and health-care providers

P4. Integration of care

Communication & information sharing for coordination & continuity of care across continuum of care

- Between healthcare providers
- Referrals to specialist
- Discharge communication
- Providing access to information and resources

P5. Family and friends' involvement and support

P5a. Involving family/friends in information-sharing and decision-making

P5b. Addressing the needs of family/friends

O1. Access to care

O1a. Timely access to care

- Wait times for referrals to see specialists, to receive a consult
- During consult, to be seen at emergency community care, pre-hospital, hospital, post-hospital; secondary care; time for patient care

O1b. Care availability

O1c. Financial burden

O2. Patient-Reported Outcomes (PROs)

O2a. Patient-Reported Outcomes Measures (PROMs)

- Health-Related Quality of Life
- Symptoms
- Functionality
- Psychosocial outcomes

O2b. Patient-Reported Experiences (PREMs)

- Recommendation or rating of hospital, health-care provider
- Assessment of care, including appropriateness and acceptability of care

O2c. Patient-Reported Adverse Outcomes (PRAOs)

- Recommendation or rating of hospital, health-care provider
- Assessment of care, including appropriateness and acceptability of care (competency, knowledge, skills of staff)

Processes

Outcomes
