# Supplementary material for: Developing a global practice-based framework of person-centred care from primary data: a cross-national qualitative study with patients, caregivers and healthcare professionals
Source: BMJ Glob Health. 2022 Jul 13;7(7):e008843. doi: 10.1136/bmjgh-2022-008843 (PMC9280875; doi:10.1136/bmjgh-2022-008843)
Supplement: online supplemental file 5 [file bmjgh-2022-008843supp005.pdf]

Appendix D. Reflexivity Log

Interviewer reflection form

Subject ID number: ________

Interviewer Initials: ________

Participant sub-group type (*circle*): patient/carer

Site: _______________ **Audio file number:** __________

Interview Date: ___/___/___

Today's date: ___/___/___

1. How would you describe the atmosphere and context of the interview?
2. What were the main points made by the respondent during this interview?
3. What new information did you gain through this interview compared to previous interviews?
4. Was there anything surprising to you personally? Or that made you think differently?
5. Were there any problems with the topic guide (e.g. wording, order of topics, missing topics) you experienced in this interview?
6. Were there points where you could have asked for more information?
7. Were there points when you could have left longer for the interviewee to respond?
8. How would you reflect upon your interviewing style? Do you feel that any of your questions may have appeared judgemental, leading or insensitive? Were any of your questions particularly good at eliciting an informative response?
