# Supplementary material for: Developing a global practice-based framework of person-centred care from primary data: a cross-national qualitative study with patients, caregivers and healthcare professionals
Source: BMJ Glob Health. 2022 Jul 13;7(7):e008843. doi: 10.1136/bmjgh-2022-008843 (PMC9280875; doi:10.1136/bmjgh-2022-008843)
Supplement: online supplemental file 6 [file bmjgh-2022-008843supp006.pdf]

Supplementary table 1. Full table of participant characteristics

**Jordan**					
Patient participants	N=50	Caregiver participants	N=20	HCP participants	N=20
Gender (Male/Female)	20/30	Gender (Male/Female)	7/13	Gender (Male/Female)	13/7
Age (years)		Age (years)		Age (years)	
Mean average (SD)	53.8 (11.8)	Mean average (SD)	41.9 (12.4)	Mean average (SD)	36.5 (8.1)
Range	26-75	Range	19-67	Range	24-55
Nationality		Nationality		Professional role	
Jordanian	28	Jordanian	10	Doctor	9
Syrian	11	Syrian	3	Nurse	11
Libyan	4	Libyan	1		
Iraqi	4	Palestinian	2	Years of experience as HCP	
Palestinian	2	Yemeni	1	Mean average (SD)	11.1 (5.3)
Yemeni	1	Jordanian of Egyptian origin	1	Range	2-22
		Jordanian of Syrian origin	1		
Interview language		Jordanian of Palestinian origin	1	Institution	
Arabic	50			Al Bashir	9
		Interview language		KHCC	11
Cancer stage:		Arabic	20		
Three	13			Years of experience working at the	
Four	36	Patient's cancer stage:		institution	
Unstageable	1	Three	2	Mean average (SD)	8.6 (5.4)
		Four	18	Range	1-20
Site					
Al Bashir	21	Site		Interview language	
KHCC	29	Al Bashir	9	Arabic	19
		KHCC	11	English	1
**South Africa**					
Patient participants	N=22	Caregiver participants	N=19	HCP participants	N=22
Gender (Male/Female)	16/6	Gender (Male/Female)	2/17	Gender (Male/Female)	9/13
Age (years)		Age (years)		Age (years)	
Mean average (SD)	57.5 (11.6)	Mean average (SD)	45.2 (14.0)	Mean average (SD)	41.5 (9.3)
Range	38-77	Range	22-69	Range	26-55
Marital status		Marital status		Professional role	
Single	5	Single	7	Doctor	12
Married	13	Married	8	Nurse	5
Divorced	4	Divorced	2	Pharmacist	2
		Separated	1	Pharmacy manager	1
Highest level education		Missing data	1	Operations manager	1
Primary School	2			Clinical manager	1
Secondary School	16	Relationship to patient			
Tertiary education	1	Daughter	7	Years of experience as HCP	
Postgraduate	1	Sister	2	Mean average (SD)	14.7 (7.9)
Post matric	2	Partner	2	Range	3-32
		Wife	1		
Religious denomination		Grandchild	1	Years of experience working at the	
Pentecostal	6	Niece	1	facility	
New Apostolic	3	Missing data	5	Mean average (SD)	6.1 (5.8)
Muslim	3				
No religion	2	Highest level education			
Methodist	1	No education	1		
Protestant	1	Primary school	2		
Anglican Church	1	Secondary School	8		
Old Apostle	1	Secondary	7		
St John Apostolic	1	College/University	1		
Other	3				
		Religious denomination			
Duration of diagnosis		Old Apostolic Church	7		
< 1 year	1	Islamic	4		
2 – 4 years	3	Methodist	1		

5 – 7 years	8	Apostolic	1		
8 – 10 years	3	Zion	1		
11- 13 years	1	Hillsong Church	1		
Not known	6	Pinkster Church	1		
		Pentecostal	2		
Other treatment received		Other	6		
No	3				
Yes – for hypertension	9				
Yes – for heart condition	2				
Yes – for depression	1				
Yes – for TB	1				
Yes - ART	1				
Yes - Cortisone inhalers	1				
Yes – for psoriasis and chronic backache	1				
Yes – for epilepsy	1				
Not known	2				
**Thailand**					
Patient participants	N=14	Caregiver participants	N=10	HCP participants	N=12
Gender (Male/Female)	11/3	Gender (Male/Female)	1/9	Gender (Male/Female)	1/11
Age (years)		Age (years)		Age (years)	
Mean average (SD)	54 (14.8)	Mean average (SD)	50.4 (8.2)	Mean average (SD)	28.7 (8.5)
Range	22-81	Range	35-62	Range	20-43
Education level		Education level		Professional role	
Primary school	2	Junior high school	1	Nurse	7
Junior high school	1	Senior high school	1	Practical nurse	5
Senior high school	1	Bachelor's degree	5		
Bachelor's degree	6	Higher than bachelor's degree	2	Years of experience working as HCP	
Higher than bachelor's degree		Vocational diploma	1	Mean average (SD)	
Vocational certificate /Diploma	2			Range	6.9 (8.1)
		Occupation			0.5-21
Occupation	2	Employee	2	Years of experience working with heart failure patients	
No occupation		No occupation	2		
Government employee		Self-employed	4	Mean average (SD)	
University employee	8	Government employee	1	Range	5.6 (7.1)
Merchant	3	Merchant/salesperson	1		0.5-21
	1				
Marital status	2	Marital status			
Single		Single	3		
Married		Married	6		
Divorced	3	Divorced	1		
Widow	8				
	2	Relation to patient			
Religion	1	Spouse	3		
Buddhism		Son/Daughter	6		
		Parent	1		
	14				
		Years of caring for patient			
		Mean average (SD)	7.5 (6.9)		
		Religion			
		Buddhism	10		

*SD: standard deviation; HCP: healthcare professionals; KHCC: King Hussein Cancer Centre; ART: Antiretroviral treatment; TB: tuberculosis
