# Supplementary material for: Developing a global practice-based framework of person-centred care from primary data: a cross-national qualitative study with patients, caregivers and healthcare professionals
Source: BMJ Glob Health. 2022 Jul 13;7(7):e008843. doi: 10.1136/bmjgh-2022-008843 (PMC9280875; doi:10.1136/bmjgh-2022-008843)
Supplement: online supplemental file 7 [file bmjgh-2022-008843supp007.pdf]

Supplementary Table 2. Illustrative participant quotations corresponding to Santana et al PCC model (2018) domains

Santana domains [34]	Illustrative corresponding quotations
**S1. Creating a PCC culture**	
**S1a. Core values and Philosophy of the organisation**	*[PCC] is possible. But it depends on the team, whether they consider this as an important issue or not.* 3002, HCP, Female, 24, Thailand
• Vision, Mission	[No corresponding participant quotations]
• Patient-directed: integrating patient experience and expertise	[No corresponding participant quotations]
• Addressing and incorporating diversity in care, health promotion and patient engagement	*[Patients] have different education and socioeconomic backgrounds. They should have some knowledge so that they can understand this kind of service system. I think that doctors, nowadays, have improved in their communication skills so they can talk to patients. It also depends on doctors' ability to apply the service. There might be a generation gap as well. I can understand this kind of service system but persons who are in the same generation as my parents may not understand and may get angry at a staff when a change is made. 1005, Patient, Male, 52, Thailand*
• Patient and healthcare provider rights	*I believe a patient has the right to know everything about their condition. ID PKN0020, Patient, Female, 42, Jordan*<br><br>*They used to make patients feel that they are entitled to every right until they die, but now it's just like any other hospital. PKH0011, Patient, Female, 44, Jordan*<br><br>*It is the family's right to be tolerated…it is [her brother's] right to ask and be answered and it is my right too. PKH0017, Patient, Male, 49, Jordan*
**S1b. Establishing operational definition of PCC**	[No corresponding participant quotations]
• Consistent operational definitions	[No corresponding participant quotations]
• Common language around PCC	[No corresponding participant quotations]
**S2. Co- designing the development and implementation of educational programs**	*They can hold educational seminars and update [doctors and nurses] on the latest breakthroughs in science in Europe and the UK. CAL0017, Caregiver, Male, 41, Jordan*<br><br>*It would be great if we…sent our staff to other countries with palliative care for courses, like the US, the UK, and France. We could have exchange programs, learn about each other's cultures, and learn about their standards and this will increase our experience in palliative care.* HCP8, HCP, Male, 30, Jordan

	It would be great if every hospital that has a heart failure clinic could exchange and share their knowledge so that they can improve the system. ID 3002, HCP, Female, 24, Thailand
**Standardised PCC training in all healthcare professional programs**	*Doctors become short-sighted when they become specialized, they ignore food. Cancer patients are not ordinary patients so they have to be tested to know if they are eating properly and not just for survival…Doctors focus on chemotherapy and forget about everything else. PKH0017, Patient, Male, 49, Jordan*<br><br>*It would be good if we as nurses had courses about the psychology of patients, especially those with advanced disease. In the first year, patients are depressed, they talk about things that are difficult to treat, but with time you become experienced in this. Nurses must know these things in the first encounter through courses. HCP2, HCP, Female, 24, Jordan*<br><br>*So if there were a programme that they could come with in discussion with psychologists with input from them as to how we could manage these sort of diseases, I think that that would useful because it would bridge the gap between knowing how to manage the patient and knowing how to understand the patient. PROF01-007, HCP, Female, 29, South Africa*<br><br>*I would like to learn more about how to encourage patients' family member to get on with their life and about what to say and do. I would like to encourage them, but I don't know what to say. I want to be able to make them feel better. ID3011, HCP, Female, 25, Thailand*<br><br>*I think we need an intensive training because talking to patients in the final stage requires an advanced skill. One wrong word can change everything. If I made them feel saddened, I would feel guilty for a long time. ID3007, HCP, Female, 27, Thailand*
• Integration of all healthcare sectors and professionals	[No corresponding participant quotations]
• Professional education and accrediting bodies	[No corresponding participant quotations]
• Translating into practice through continued professional education and mentorship	*We need more accessible training courses whether it be within Jordan or abroad because we must stay updated with the newest guidelines regardless of subspecialty.* HCP1, HCP, Male, 41, Jordan<br><br>*Something good about the hospital is the continued medical education courses, which allow lots of lucky nurses to continue to increase their scientific knowledge until they reach a level where their knowledge is adequate. HCP3, HCP, Male, 40, Jordan*<br><br>*There haven't been any courses organized by the ministry or the hospital for palliative care. Two years ago, there was a course on oncology I organized, that no one from the ministry would set up. Do you understand? I'm a head nurse and I'd like to make changes to the administration so we could bring external lecturers. However, if you want to bring external lecturers, we need to pay them and we can't afford to.* HCP11, HCP, Female, 35, Jordan<br><br>*I think in this hospital we have got quite a good network if you are stuck there is lots of help…We have got quite a lot of senior colleagues here. I am fairly junior here and you can always just come and look at this patient what do you think, you know why is there is something that I am missing. PROF01-003, HCP, Female, 31, South Africa*

**<u>S3. Co- designing the development and implementation of health promotion and prevention programs</u>**	*I asked Dr X and he told me to eat anything I want but to stay away from sugar…I follow the doctor's orders. An ounce of prevention is worth a pound of cure.* PAL0039, Patient, Male, 71, Jordan<br><br>*There is also an increased tendency of smoking among smokers…It doesn't matter how much to ask them to cut down, it has to do more with the psychological aspect – they're trying to blow off steam by smoking. HCP13, HCP, Male, 35, Jordan*<br><br>*I have tried [to quit smoking] but I cannot, that is the whole point, I cannot because I am stressing too much and we have got a lot of things on our mind because that makes you stress. PAT03-006, Patient, Female, 52, South Africa*<br><br>*I think we treat the exacerbations quite well…. but I think treating the patient as a whole and addressing the small things they could probably make a difference in their disease progression like the smoking, inhaler techniques all of those sorts of things. I think we are probably missing the boat at the moment with that. PROF01-003, HCP, Female, 31, South Africa*<br><br>*I think that prevention and education around smoking is the most important thing, which is very difficult with our clientele. I think in any socio-economical group it is quite difficult and I feel that it is quite important to see why patients are smoking and it goes around anxiety a lot of the time and I don't think that we are always focusing on how to help the patient stop smoking. We just tell them they should but I do think that helping their anxiety and how to deal without smoking is quite important.* PROF01-004, HCP, Female, 41, South Africa<br><br>*Most of us work in a very pressured environment. So, it is quite difficult if you have to take a page and draw the lungs and explain to patients what is happening, why they are getting sick. I think they should have a better health promotion option like, they should in the waiting areas have videos playing explaining, establishing support groups at the hospital maybe by one of the Sisters in the OPD or train somebody that can do a talk once a month that we can refer the new patients to. PROF01-008, Female, 26, HCP, South Africa*<br><br>*If the general public were made aware of how heart disease develops, it would be great so they could take care of themselves well. Later, the doctor should inform the public via public media on best practice of personal care for heart disease patients. I've seen some heart disease patients still drinking alcohol without taking care of themselves after heart surgery; they said they didn't know how to take care of themselves because the doctor told them nothing despite the hundred thousand expense on the operation. 1011, Patient, Male, 61, Thailand*
**S3a. Collaboration and empowerment of patients, communities and organisations in design of programs**	[No corresponding participant quotations]
• Identify resources	[No corresponding participant quotations]
• Creating partnerships with community organisations	*So, we can link with NPOs, we can do home visits for patients who, they need to be identified and say 'this is a high-risk patient, he is struggling'. So, we can do home visits for the NPOS, that is what is called …there is a programme called COPC that is coming into effect, Community Oriented Primary Care. So, that is basically where they are looking at missed people. So, let us say there is ten people in the house but only the mother and the father come and access healthcare. What of the other eight people in the house? Chances are they have got some healthcare conditions that need to be looked at. PROF07-005, HCP, Male, 47, South Africa*<br><br>*But I think on-going teaching as we need and we are also getting support from other NGOs and other companies who are dealing with COPDs and asthma so they do come to give some talks as well and also assist in terms of the techniques for pumps and breathing. PROF08-001, HCP, Male 52, South Africa*

	It would be good if we can have more home visits and more collaboration with local hospitals. We already advise patients who don't have a blood pressure meter at home to let a volunteer villager help them monitor their blood pressure. 3002, HCP, Female, 24, Thailand<br><br>*He has lived in a temple and has done some volunteer work there for 18 years. When he gets sick, he can ask for help from people living in the temple. He can also get his meals from the temple. 2006, Caregiver, Female, 62, Thailand*
• Create patient advisory groups	[No corresponding participant quotations]
**<u>S4. Supporting a workforce committed to PCC</u>**	
**S4a. Ensure resources for staff to practice PCC**	
• Provide adequate incentives in payment programs; celebrate small wins and victories	*They are supposed to motivate the staff to improve their skills, even if it was a financial motivation.* PKH0025, Patient, Male, 69, Jordan
• Encourage teamwork and teambuilding	*By dividing the roles between the medical staff everything would be easy. I mean it is hard to bear all the responsibility by yourself only, you need help from others. So when this is applied, everyone will take his or her right in this responsibility.* HCP16, HCP, Male, 35, Jordan<br><br>*I feel they don't have control over their inferiors, like nurses. Every nurse walks around like she owns a place and they don't stand each other. They don't get along…Nurses don't dare tell the housekeeping to clean the rooms or take out the trash or clean the toilet. There is no cooperation. PKH0030, Patient, Female, 33, Jordan*<br><br>*With some staff we are able to discuss, what does it mean, how you feel about this, what has frustrated you, between the doctors and the nurses and the doctors and other doctors and then between the doctors and specialists. I think there are supportive relationships, I think there could be ways in which they could be more supportive potentially but I am not sure exactly how or how that would look. Maybe create another space where we can discuss. PROF01-005, HCP, Male, 32, South Africa*<br><br>*We have collaborated for a long time; we get along well including the doctor, nurse, and pharmacist and we understand each other. Having a personal bond helps us collaborate well, that is, our communication runs smoothly, and we work with no pressure.* 3010, HCP, Female, 43, Thailand<br><br>*We can consult other specialties when we have problems. We can also communicate with a doctor or a pharmacist directly. We can ask a doctor when we need more information or would like to know his opinion regarding patient's symptoms. 3002, HCP, Female, 24, Thailand*
**<u>S5. Providing a supportive and accommodating PCC environment</u>**	[See subdomain quotations]
**S5a. Designing healthcare facilities and services promoting PCC**	[See subdomain quotations]

• Collaborate with and empower patients and staff in designing healthcare facilities	[No corresponding participant quotations]
• Environments that are welcoming, comfortable and respectful	*Hygiene is terrible…beds are disgusting, bathrooms are terrible and there is no water, no sanitizers, and the smell.. I would run out of the room and sleep outside. I was afraid to sit on the chair or sleep in my room.* PKH0030, Patient, Female, 33, Jordan<br><br>*It is tiring to be honest, I once tweeted that you should be a class-A athlete when you come here. I have to go get the pharmacist's signature, then I have to go get a couple of signatures from archiving and the secretary, then I have to go to the delivery ward too and it is far away.* CAL0016, Caregiver, Female, 28, Jordan<br><br>*We were made to stand outside and queue. To queue a queue there outside. A line there outside. Then again we are taken inside there in the veranda while feeling cold …feeling cold there outside.* PAT03-003, Patient, Female, 67, South Africa<br><br>*One main concern is the congestion. Everything is so cramped up and it frustrates people and that is where the arguments start and sometimes where the fights starts.* PAT03-008, Patient, Female, 50, South Africa<br><br>*Some days the seats are not enough for patients. Sofas are too soft so patients who are the elderly and those who are exhausted may find it difficult to get up and stand. 3002, HCP, Female, 24, Thailand*
• Spaces that provide privacy	*It is the atmosphere in the hospital, I might be doing well, but just having a patient in pain with me in the same room is enough to make me feel terrible, so each patient should have their own room and not witness others' pain.* PAL0046, Patient, Male, 49, Jordan<br><br>*See we are all in one room. There is a table. All the people sit there. Now they ask, 'do you have' and everybody hear your complaints. Everybody can hear if something is wrong with you or so then I just say, nothing is wrong because I do not want to explain with a room full of people.* PAT07-005, Patient, Female, 45, South Africa
• Spiritual and religious spaces	*We have a copy of Quran here. PAL0004, Patient, Female, 46, Jordan*<br><br>*[No corresponding participant quotations]*
• Facility that prioritise the safety and security of its patients and staff	*The reason why I am attending here is because that other hospital is situated in gang land. The gang comes in there when there is a shooting there and they come after the one that they shot, then you must all be hiding. Now, how on earth can you feel free or safe to go like operate like that?* PAT01-005, Patient, Male, 69, South Africa<br><br>*They are at risk of infections in the hospital so that is why if you have a separate suite that was just for the COPDs and asthmas, you don't have the TBs floating in there and making them ill.* PROF01-002, HCP, Male, 45, South Africa<br><br>It is not crowded unlike the outpatient unit of the internal medicine department…there are so many people standing around us. It is such a bad situation, especially during the COVID-19 pandemic situation. We need to do physical distancing but we can't since every patient has their relatives with them. *2005, Caregiver, Female, 50, Thailand*
• Areas/rooms that will support the accommodation of patients	*We have got a plenty of space and our place is very clean. It is not so crowded here so patients and their relatives are quite okay to wait for a doctor.* 3002, HCP, Female, 24, Thailand

**S5b. Integrating organization-wide services promoting PCC**	*[See subdomain quotations]*
• Provide interpretation and language services	*We're still Arabs and we can understand one another. As for those we can't understand, like foreigners, we have a list of translators, who either speak the language from living in other countries or from learning it. HCP8, HCP, Male, 30, Jordan*<br><br>*I don't actually speak the language of my patients, my Arabic is very poor, especially with palliative care I could do basic symptom assessment, but the richness of the consultation I am relying on my team members for translation. Sometimes, somethings are not translated in a way that is gentle as need to be. I think this is another barrier for providing good palliative care.* HCP10, HCP, Female, 40, Jordan
• Patient-directed visiting hours	[No corresponding participant quotations]
**S6. Developing and integrating structures to support health information technology**	[See subdomain quotations]
**Common e-health platform for health information exchange across providers and patients**	*They know everything, they can access my file from the system just by me giving them my number.* PKH0033, Patient, Female, 53, Jordan<br><br>*I would like to be able to use technology to send information about my symptoms and blood pressure to the hospital so that the doctor can recommend what I should do. 1008, Patient, Male, 63, Thailand*<br><br>*Normally, a doctor will order us to do telemonitoring with patients after they visit our clinic... Patients will take a picture of their records and send it to us via the official LINE of the heart failure clinic.* 3004, HCP, Female, 25, Thailand<br><br>*So when I came to the emergency room, they looked in a computer and just asked for more information. They already knew some of my medical information. I think the process here works well.* 1005, Patient, Male, 52, Thailand<br><br>I'm not good at communicating via the Internet like this clinic does as I'm old-school and not good with computers. 1007, Patient, Male, 60, Thailand
• Electronic Health Record systems with capacity to coordinate and share healthcare interactions across the continuum of care	*Now it is easier because it is all computerized. They can check the result on the computer. Even requesting a scan or booking an appointment…it is a little better with the computer system.* PAL0040, Patient, Female, 43, Jordan<br><br>*We used to bring the patient's file to another hospital, but the handwriting was difficult and some was in English. The doctor could not understand and had to ask many questions for medical history, but we could not remember it all. I believe if the medical history can be stored in a thumb drive which we can bring with us anywhere, that would be helpful, especially if the patient has a long and complicated medical history.* 2009, Caregiver, Female, 45, Thailand
• Health information privacy and security	[No corresponding participant quotations]
• E-health adoption support through strategic funding and education	*[No corresponding participant quotations]*

**S7. Creating structures to measure and monitor PCC performance**	*There is this sense of hopelessness that you see these whole bunch of inhalers being returned and 20% of them are still full and you just realise that we are so busy with just getting the numbers out that there is no one-on-one dealing with the individual. We are just looking at the whole group as a whole and just trying to get the numbers out and it is not getting to the root of the problem. And at the end of the day you realise that that is the importance of having protocols and standard operating procedures and you end up putting the whole situation into a box and you need to operate out of that box. And I think…a lot of patients are slipping through the system because of this.* PROF01-010, HCP, Male, 55, South Africa
**Co-design and develop framework for measurement, monitoring and evaluation**	[No corresponding participant quotations]
• Co-design and development of innovative programs to collect patients and caregiver experiences about care received and providing timely feedback to improve the quality of health care (including complaints and compliments, wins and lessons learned)	[No corresponding participant quotations]
• Reporting and feedback for accountability and to improve quality of health care	[No corresponding participant quotations]
**P1. Cultivating communication**	[See subdomain quotations]
**P1a. Listening to patients**	Listening is one way of showing respect - *they listen to whatever I say. They understand my needs and they do as I request…They respect me and value me. PAL0007, Patient, Male, 68, Jordan*<br><br>*They like to be heard. It may be more important to hear the patient out and make sure they're satisfied, than to give them medication. HCP4, HCP, Female, 31, Jordan*<br><br>*I think they must use their ears sometimes. And listen. They must really listen to another person's needs. CAR03-008, Caregiver, Female, 39, South Africa*<br><br>*I would have to say is the tendency is not to listen. The tendency is to say, to go to the mental filing cabinet and pull out the piece of paper that says COPD tick off 1,2,3, 4,5, this is how you treat CPOD, bang, bang, bang, and you don't actually listen to them. You listen to the patient when what are the patient saying fits within that list. PAT01-001, Patient, Male, 67, South Africa*<br><br>*I think that talking is good for me, as in asking about my living and suggestions on personal practice, this would give me courage. Asking about my life will make me feel at home. 1007, Patient, Male, 60, Thailand*
• Gathering information through active listening	*When you first meet these patients, they're very secretive and quiet, since we don't live in a society that allows these patients to express themselves. When the patient starts to trust their care providers, they start to talk about their thoughts and worries…If the patient doesn't mention anything, we'll ask them. Patients are like books and you have to open them up and read the pages in depth. HCP3, HCP, Male, 40, Jordan*

	Interviewer: How do you feel that this care could be made better? Participant: I suppose listening to people and see what they have to say…Finding out what their needs are and how they can be fulfilled, which seems to be exactly what we have just done. PAT01-003, Patient, Male, 61, South Africa<br><br>*Instead of probing and asking me the relevant questions to take a decision on what to do, they naturally assumed that the crisis that we were having was acute and they instantly deferred us to the Critical Care Unit…Had that individual doctor asked the relevant question then he would have known that, that situation was not acute. CAR04-002, Caregiver, Female, 26, South Africa*<br><br>*He used to ask other information about her life. I think this is okay. 2007, Caregiver, Female, 62, Thailand*
• Asking questions of what patients want to discuss (concerns, views, understanding)	*Nurse X and Nurse Y are great and helpful. You should see the conversation between me and X, she always urges me to ask questions and comforts me. PAL0044, Patient, Female, 50, Jordan*<br><br>*I like to ask a lot of questions and he always tells me I am doing fine. I know he doesn't want me to ask questions…Even if he clarifies he does it minimally: "Your condition is stable; you have to keep on taking chemo". PKH0020, Patient, Female, 42, Jordan*<br><br>*If you ask for something, they will advise you…They will not push you aside. They will always have an answer if you do ask a question, which is always in a good way. PAT07-003, Patient, Male, 73, South Africa*<br><br>*I like the doctor gives me opportunities to ask questions. 1014, Patient, Male, 41, Thailand*<br><br>*The patient also might not know the important things they need to know or the options they have. By giving them the information, you're putting the ball in their court. HCP06, HCP, Female, 31, Jordan*<br><br>*The doctors don't even really speaks so a lot. And sometimes my mommy is in such a lot of pain and you can see it's just, just sitting here and she doesn't even say a word. CAR03-002, Caregiver, Male, 28, South Africa*
• Non-verbal behaviours (eye-contact, listening attentively, proximity/touch, head nodding)	*They are always smiling, which is the most important thing. It is comforting. PAL0043, Patient, Male, 50, Jordan*<br><br>*When I was talking to him, he was turning his back to me, so what do you expect me to read from his body language? PKH0017, Patient, Male, 49, Jordan*<br><br>*I came in with my mother and they told me that I could not come in. So I just walked past them and they did not even smile. CAR03-012, Caregiver, Female, 30, South Africa*<br><br>*The patient used to be admitted to another hospital and the doctor stood so far away from us. It made us feel like he was afraid that we would spread a germ to him. 2006, Caregiver, Female, 62, Thailand*
**P1b. Sharing information**	*I like to ask a lot of questions and he always tells me I am doing fine. I know he doesn't want me to ask questions…Even if he clarifies, he does it minimally. "Your condition is stable, you have to keep on taking chemo then the hormonal therapy". PKH0020, Patient, Female, 42, Jordan*

	If you ask for something, they will advise you, they will tell you what to do, what not to do. They will not push you aside. They will always have an answer if you do ask a question, which is always in a good way. PAT07-003, Patient, Male, 73, South Africa<br><br>*I feel better even if I cannot fully understand. I will learn about the basic information about how to take care of myself. It takes me a while to fully understand them. At first, I did not quite understand, but the doctor tried to explain. 1010, Patient, Female, 60, Thailand*
• Patients are provided with all the necessary information to make informed decisions in relation to their diagnosis and treatment plan	*I wish they would tell me everything and not keep anything from me so that I can continue with my treatment. I am not afraid of it. I must know everything and get the treatment because I have 8 children to take care of. PAL0037, Patient, Female, 45, Jordan*<br><br>*I was surprised after the surgery when I found out the whole stomach was removed, they had told me they would remove a part of the stomach, not all of it, they didn't even say they would remove half of it…I was shocked, and I was a mess, I got very upset and sad. PAL0046, Patient, Male, 49, Jordan*<br><br>*The only person who has ever really spoken to me about with any kind of sense was a guy called Dr X … for 20 minutes he talked to me, explained to me what was going on and why…the values of some of the different medications or the limited values of some of the different medications. Uhm and that was really eye opening and enlightening in that as a patient sometimes you get wrong perceptions. Because you base your perceptions on half words and half sentences because the doctors are not talking to you, but they really talk to the nurse or whoever, but of course you're listening. PAT01-001, Patient, Male, 67, South Africa*<br><br>*I want to know in details what effects the medicines have. Normally, he tells us how each medicine works such as decrease hypertension, decrease body swelling and prevent blood clot. But I don't know whether it is necessary to take this large amount of medicines or not. I give the medicines to my dad without knowing in details. I really want to know if all medicines he is taking are necessary. 2005, Caregiver, Female, 50, Thailand*<br><br>*The doctor should tell the patient the truth, informing them how future symptoms and treatment plans will be so that the patient can make a plan to adjust themselves. If I were a patient, I definitely wouldn't want the doctor to keep it to themselves. 3012, HCP, Female, 30, Thailand*
• Sharing of information regarding patient's condition and their own impact/influences on their condition	*I want the doctor to talk to me about my condition, I don't like it when he's vague. I want to know what I can eat, what stage I am at, what my current situation is…The doctor stops by in the morning, asks me how I am doing and then leaves. PAL0003, Patient, Female, 47, Jordan*<br><br>*The words you use with your patients are very critical, when you tell a patient their disease is very "aggressive", that means they won't tolerate it, so by the time I walked out of the clinic, I was a mess. A patient shouldn't hear the word "aggressive" … it is nice to have a smart doctor who knows when to tell and how much to tell. PKH0017, Patient, Male, 49, Jordan*<br><br>*As doctors we don't spend enough time with our patients explaining to them this is what is wrong with you, this is what you can expect, these are the improvements that you can expect, this is what happens when you are unwell. I think if patients understand what it is that is wrong with them it will help them to cope with their illness better. PROF01-003, HCP, Female, 31, South Africa*<br><br>*If I know how my symptoms will progress and what the treatment procedures are, it makes me feel good...I don't have the knowledge, so I get curious and worried. 1013, Patient, Male, 50, Thailand*<br><br>*We need to improve our approach in terms of asking people of how much information they want. HCP10, HCP, Female, 40, Jordan*

	The doctor should ask first if the patient wants to know or not. If he wants to know, the doctor can gradually give him information. If he does not want to know and the doctor tells him, he will be very worried. 1010, Patient, Female, 60, Thailand
**P1c. Discussing care plans with patients**	*I want the doctor to tell me what stage I reached and to what extent I benefited from the treatment and how much treatment I have left. I want to know what they're going to do next or what they're planning to do if I'm cured. PAL0003, Patient, Female, 47, Jordan*<br><br>*I think we need to improve our approach in terms of asking people of how much information they want to be given, and also discussing things like preferred place of care, preferred place of death and other preferences for the future. What kind of care they would want, and what would they not want. We need to do what we call advanced care planning. HCP10, HCP, Female, 40, Jordan*<br><br>*An advanced care plan. I think that will be something good. PAT07-001, Patient, Male, 73, South Africa*<br><br>*To know more about the COPD and what is the road ahead. If she is just going to slowly get worse. If she is going to suddenly stop breathing. I am not sure about how she is going to be ahead. CAR01-002, Caregiver, Female, 45, South Africa*<br><br>*If I know how my symptoms will progress and what the treatment procedures are, it makes me feel good. That way, I know how the treatment will be. I don't have the knowledge, so I get curious and worried. 1013, Patient, Male, 50, Thailand*<br><br>*I think [discussing the treatment plan] one stage at a time is better…Because all I care about is for the pain to go away. I don't really care about the disease. PKH0011, Patient, Female, 44, Jordan*<br><br>*I don't know what I need to know about the future. So I think I would rather focus on the present. I am not sure whether I will get more anxious or not if I know about the future symptoms. 1006, Patient, Female, 58, Thailand*<br><br>*I don't want to talk about a long-term plan. I just live my life day by day. 1001, Patient, Male, 44, Thailand*<br><br>*Usually, doctors would tell patients only about their current condition because if they talk about the future, patients would be discouraged and wouldn't want to continue the treatment.. 3011, HCP, Female, 25, Thailand*
• Responding to patient and caregiver needs	*They keep forgetting that my body is different than their bodies. My blood is different than their blood. A lot of things are different. You might take a morphine pill and feel better but I'm taking 240 mg and I'm not feeling any better. They're not taking this point into consideration. PKH0013, Patient, Male, 68, Jordan*<br><br>*They listen to her and what she needs. The nurses are very good but my mom also helps herself. So, if she needs something she will get up and say I need this. CAR01-002, Caregiver, Female, 45, South Africa*<br><br>*[Further data corresponded to this domain are listed under relevant more specific domains]*
• Aim and follow-up of treatment or interventions with possible outcomes and adverse events/side-effects	*I believe doctors should tell patients, especially cancer patients, every single detail of their condition. When I…started taking hormonal therapy I used to get my periods, then it stopped in July, and when I asked the doctor about it he told me I might never get it again, and they hadn't told me that before. How was I supposed to know? PKH0020, Patient, Female, 42, Jordan*

	I was prepared for the hair loss, it is different when you're caught by surprise, this is what upsets a patient. PAL0040, Patient, Female, 43, Jordan<br><br>*If I knew [about the potential side-effects] I wouldn't have taken it or might have experienced additional side effects…Because you will start thinking about it, and the more you think about it, the more likely it is to happen. PAL0044, Patient, Female, 50, Jordan*<br><br>*Every time I see the doctor, I would ask him whether the medication produces any side effect. 1013, Patient, Male, 50, Thailand*<br><br>*Patients usually misunderstand that surgery would make the symptoms completely go away. They don't understand that it's only one step of the treatments and there would be more treatments. 3011, HCP, Female, 25, Thailand*<br><br>*I think I would rather focus on the present. I am not sure whether I will get more anxious or not if I know about the future symptoms. 1006, Patient, Female, 58, Thailand*
• Discussing and building capacity of patients for self-management and self-care	*Patient education helps the patient get ready to go home without having to constantly return because they don't know how to deal with certain things. This all depends on how involved the patient is in their education. HCP12, HCP, Female, 28, Jordan*<br><br>*I would like to do now is for me to take more care of my health. My physical health and be more serious about it as I am growing older and I need to take care of myself. PAT03-008, Patient, Female, 50, South Africa*<br><br>*Patients have no idea how they are using their inhalers. We saw someone in trauma yesterday, mouth open just squirted into their mouth. PROF04-002, HCP, Female, 29, South Africa*<br><br>*The doctor allows me to adjust Lasix intake by following the instructions advised on the adjustment…The fact that I could adjust my medication enabled me to look after myself at home. If my condition was to get a bit worse, I would increase the dose. If I urinated too much, I would lower the dose. 1007, Patient, Male, 60, Thailand*<br><br>*I think when they know how to cope with their symptoms and how to solve the problems, they may feel relieved from stress. 3001, HCP, Female, 26, Thailand*
• Acknowledging and discussing uncertainties	*She didn't expect [relapse] especially because she didn't know it carries a high chance of recurrence. CKH0008, Caregiver, Female, 31, Jordan*<br><br>*It is unpredictable thing. You don't know when your patient is going to get ill or when you discharge them are they going to be back tomorrow because they are suddenly feeling short of breath that lack of control I think often leads to a sense of being a bit despondent when you are treating these patients PROF01-003, HCP, Female, 31, South Africa*<br><br>*The staff at the Centre for Organ Transplantation told me that finding a compatible heart is not easy. Some people wait for only a few weeks. Others had waited for three or four years before they died without the transplant. Waiting for a heart is like buying the lottery. 1001, Patient, Male, 44, Thailand*<br><br>*[The doctor] told me that it is difficult to predict the future since heart disease is different from cancer that has a disease stage. 2002, Caregiver, Female, 53, Thailand*

• Creating a shared understanding	*Other patients need the information to be repeated multiple times when we can't get the idea across. HCP7, HCP, Female, 27, Jordan*<br><br>*There were some words that would be understood differently or would have a different meaning in their dialect. We also faced this difficulty as nurses and doctors, mostly with the Libyan patients…We would ask them what exactly certain words mean to make sure that we understood correctly and vice-versa, we'd explain words to them. HCP7, HCP, Female, 27, Jordan*<br><br>*But it is just sometimes they use this doctor version words like COPD and things that I do not understand sometimes. They must, how can I say to you, they must give the normal version… they must use simple language which the family member can understand. CAR03-008, Caregiver, Female, 39, South Africa*<br><br>*I'd like him to use simple language not medical terms. I can never remember the name of medicines since their names are unfamiliar…I also need simple explanations on how organs work. Using medical terms makes me confused. 1005, Patient, Male, 52, Thailand*<br><br>*Use simple language. Make comparisons to common things in the patient's daily life. 2008, Caregiver, Female, 35, Thailand*
**<u>P2. Respectful and compassionate care</u>**	*[The HCPs] are nice, but if we're talking respect, they should give me adequate time, explain my condition to me, and be able to handle my questions. PAL0046, Patient, Male, 49, Jordan*<br><br>*They are all very respectful. I respect them and therefore force them to respect me. It is all up to the person you're dealing with, I mean, if I was to insult one of the staff at Al-Bashir or Istishari, he would insult me back, right? CAL0013, Caregiver, Male, 55, Jordan*<br><br>*I would like, they have to talk professionally, I mean, you are doctors, so you must act like one, respect like I respect the doctor, the doctor must respect the patient and, yes….To talk in a good manner, not to shout at you or maybe your face expression, they say your first expression is important.CAR03-006, Caregiver, Female, 22, South Africa*<br><br>*Today, a doctor asked me about my career and my position at work. This made me feel good. In the past, I was perceived as an ordinary person but now…as they know that I am a lecturer, they change the way they speak with me. 1005, Patient, Male, 52, Thailand*
**P2a. Being responsive to preferences, needs and values**	*The most important thing is that they keep checking on the patient. CAL0002, Caregiver, Male, 36, Jordan*<br><br>*We ask them what bothers them physically, whether it was pain, appetite problems, or shortness of breath. We then move to psychological issues, we ask them how they feel, then we ask about spiritual issues. HCP2, HCP, Female, 24, Jordan*<br><br>*I suppose…[HCPs should] find out what their needs are and how they can be fulfilled which seems to be exactly what we have just done. PAT01-003, Patient, Male, 61, South Africa*<br><br>*We try to help in whatever way we can. For example, if a patient is hungry, our will ask our housekeeper to buy food for them. 3003, HPC, 42, Thailand*<br><br>*There should be an assessment of individual patient's specific needs because individual patients have different needs. 3008, HCP, Female, 42, Thailand*

• Acknowledge the patient as an expert in their own health and as a part of the healthcare team	*They had calculated my dose according to my previous weight, which is 70 kg, but my weight at the time of the cycles was 55. I told them it was making me tired, but no one would listen... I could've avoided reaching this stage. PKH0011, Patient, Female, 44, Jordan*<br><br>*He asked the [trainee] doctors if they know what the diagnosis was, and they wouldn't know, so he asked me to explain my diagnosis to them to make me feel special. He was like "explain your disease to the doctors". That made me feel I am a survivor and that I should be very proud of myself. PKH0017, Patient, Male, 49, Jordan*<br><br>*Some of them they have been living with COPD for a very long time so which is also very important to sit down with them as well just to find out what do they think as healthcare workers we can do to support them. PROF08-001, HCP, Male, 52, South Africa*<br><br>*Patients who have just been diagnosed with heart failure are eager to receive treatments because their symptoms are only in the initial phase and they want to get better and get back to normal. They will strictly follow advice. 3007, HCP, Female, 27, Thailand*
• Understanding patient within his/her unique psychosocial or cultural context (i.e: awareness of religious, spiritual, lifestyle, social and environmental factors)	*It is God who will cure me, I always ask him to strengthen me, and that's when I feel that he loves me, I like to think that he is putting me through this as a form or redemption from my sins or to get me closer to him. PAL040, Patient, Female, 43, Jordan*<br><br>*I think in terms of spiritually when people start realising that they are not going to live very long. And as health professionals we don't often have those conversations although in palliative care we should. PROF07-002, HCP, Female, 49, South Africa*<br><br>*The patient pays homage to a Buddha image and prays before bed every night; she then feels better and calmer. 2010, Caregiver, Female, 52, Thailand*<br><br>*I seem to be opposed to religion of all kinds because in my mind's eye I see it as brainwashing. That also causes major fear because it is so prolific. PAT01-003, Patient, Male, 61, South Africa*<br><br>Religion is not the patient's spiritual anchor. His spiritual anchor is his mother who always encourages him. 2003, Caregiver, Female, 43, Thailand
• Responding empathically	*The patient next to my sister asked a nurse to raise the pillow for her because this patient is not accompanied, and the nurse said "Did you call me just for this?". I went and complained to the nurse in charge. CKH0019, Caregiver, Female, 40, Jordan*<br><br>*[HCPs should] find out what their needs are and how they can be fulfilled which seems to be exactly what we have just done. PAT01-003, Patient, Male, 61, South Africa*<br><br>*We try to help in whatever way we can. For example, if a patient is hungry, our will ask our housekeeper to buy food for them. 3003, HPC, 42, Thailand*
**P2b. Providing supportive care**	[See subdomain quotations]
• Building a partnership with patients	*There should be mutual trust between the patient and the doctor, and the doctor's attitude is also important. PKH0025, Patient, Male, 69, Jordan*

	If I'm not convinced by the doctor, treatment is useless. If you don't like your doctor you won't benefit. If he was a GP and you liked him, that's 80% of the treatment. PAL0010, Patient, Male, 65, Jordan<br><br>*It takes time. It is about building up trust with that person, to make them understand that they are important, and you intend to do what is best for them. When they know that they are important, and that you are going to give them the best care, people will usually accept the options. HCP10, HCP, Female, 40, Jordan*<br><br>*I want to come here. I trust the team here. 1006, Patient, Female, 58, Thailand*
• Providing resources	*[Data corresponded to this domain are listed under more specific relevant domains]*
• Sensitivity to emotional/psychosocial needs	*Anything makes me upset, I don't have tolerance…I feel very angry, I can't tolerate anything… [I am anxious] because I am alone here, no father, no family. PKH0036, Patient, Female, 31, Jordan*<br><br>*Honestly I am afraid of chemotherapy, and every time someone mentions chemotherapy I feel fear…when chemo is mentioned, I don't accept it and neither do I accept my disease. PAL0001, Patient, Female, 45, Jordan*<br><br>*There are patients who do ask questions regarding depression and social issues, we face this frequently…Honestly for palliative patients we like to deal with every aspect because sometimes patients tell you they're in pain but the source is social or spiritual so the patient improves when you don't do something medication related but something else. HCP7, HCP, Female, 27, Jordan*<br><br>*I suppose I live in a kind of frustrated fear, anxiety and depression. PAT01-003, Patient, Male, 61, South Africa*<br><br>*You sort of have these two interplaying with each other - the COPD that causes breathlessness, that makes me anxious, my anxiety that increases my breathlessness and then they sort of feed into each other. PROF01-005, HCP, Male, 32, South Africa*<br><br>Having a sense of control and self-management can reduce anxiety and other psychological challenges - *She can adjust herself to the disease. She knows how to take care of herself so she does not feel anxious. 2007, Caregiver, Female, 62, Thailand*<br><br>*Mental health care is yet be taken seriously…. Most patients are worried about cardiac arrest. 3012, HCP, Female, Thailand*<br><br>*At first, we paid from our own pocket, then my husband drained all his money…Doctor X, he told us about the Goodwill Fund and we suddenly ran into a doctor who is a manager here and he told us to apply. Dr X wrote the application. PKH0033, Patient, Female, 53, Jordan*<br><br>*The patient is covered from A-Z, with every patient coming into the Centre being seen by a social worker as part of the screening process, where we ask about their caretakers, financial status, and social problems. HCP8, HCP, Male, 30, Jordan*<br><br>*Then people will just tell you that, "I don't need to talk. All I need is this and that. I need to work. I need employment. I need food. PROF03-003, HCP, Female, 35, South Africa*<br><br>*Needy patients generally have difficulty with travel costs, so we send them to the department of social work for financial assistance…They were happy about it; we assisted them to the best of our abilities. We booked appointments for them as much as necessary. 3021, HCP, Female, 20, Thailand*

	If they have social problems, we will consult a social worker. We help them to contact a social worker. 3004, HCP, Female, 25, Thailand<br><br>*I like to sit with a friend or my brother and talk because they are close to me, maybe if I talk to a therapist, I might shy out. I know I tell the doctor about everything, but something I am just too shy to say to someone who is not a friend, I like to hear what older people think. PAL0043, Patient, Male, 50, Jordan*<br><br>*There is a culture of shame towards psychological care, but some families understand that the patient has gotten to the point where they need this help and it's as important as the rest of their treatment. HCP18, HCP, Male, 54, Jordan*<br><br>*I do not expect as I know doctors do not get emotionally involved with their patients. That is not like a whole friendly thing it is more of a business relationship. He is doing his job and I am the subject. PAT01-003, Patient, Male, 61, South Africa*<br><br>*I don't want to bother him/her and don't want to be difficult to deal with. The doctor is not a social worker; it's not his/her responsibility to solve my personal issues. The doctor is in charge of treating the ill merely. 2010, Caregiver, Female, 52, Thailand*
**P3. Engaging patients in managing their care**	[See subdomain quotations]
**Co-designing care plans with patients**	*He told me not to hurry and that I ask too many questions, and they would provide me with information one step at a time…I get to know how to live my life and what my treatment plan will be, since I am here for a month and a half, I am not an employee and I don't have a salary, so me being here affects my life at home, they don't have someone to provide for them…I asked him because I wanted to plan ahead, and they didn't give me the right information. PAL0046, Patient, Male, 49, Jordan*<br><br>*The health service as it stands at this stage provides a basically what is an emergency stop-cap service …what I believe you need, with this particular disease is you need a plan…you need to, to be able to say, this treatment isn't for now this treatment is for ongoing. PAT01-001, Patient, Male, 67, South Africa*<br><br>*I think when you engage with a patient and you allow them to have an opinion and allow them to have a say, it is very liberating for both you and them because you do not feel like you are just handing out some orders and there is also a feeling that if they actually want to do this, if this is coming from them then they probably going to carry through with this. PROF01-007, HCP, Female, 29, South Africa*
• Shared decision making	*It should be a joint decision…It is possible that the doctors want to do something that I don't approve of. When it was time for my sixth cycle, I told Dr X that it is making me ill and it is damaging my immunity, so he told it was ok if I didn't want to take it. PKH0023, Patient, Male, 55, Jordan*<br><br>*Most of the time they will listen, they will respond, we will make a decision together. CAR04-002, Caregiver, Female, 26, South Africa*<br><br>*The patient and I greatly took part in decision making; the doctor gave us information and let us opt in or out…It's good because the surgery has risks and the patient will feel that she chooses to accept the risks…. Collaboration is likely to bring about the best treatment method. 2010, Caregiver, Female, 52, Thailand*

	I am not a doctor…I believe the doctor should just inform the patient about the plan because the doctor knows what's best for the patient. People tend to get emotional and they might freak out and decide against chemo just because they don't want to lose their hair or lose weight. PKH0021, Patient, Female, 26, Jordan<br><br>*I tend to just tell the patient, "We are going to keep the medication same, everything looks good, are you happy?" And then most of the time they will say, "No it is fine," because they feel fine. PROF04-002, HCP, Female, 29, South Africa*<br><br>*I would like the doctors to decide because they can do it better. They have studied medicine, so they know what to do. I am just a patient, and I don't know anything. It's better to let doctors decide. 1012, Patient, Male, 72, Thailand*<br><br>*In the western world, the patient comes into the doctor's office with more knowledge on their condition than the doctor does. They come in ready to discuss whatever they want to know. The patients here come in and put the decision in the hands of their family, which their family puts in the hands of the doctor.…you share the facts and what you know with the family so they can make a decision and they just tell you to do whatever you feel is right. HCP18, HCP, Male, 54, Jordan*<br><br>If the patient doesn't know anything, we'd have to make the decisions with the family. HCP7, HCP, Female, 27, Jordan
• Goal-setting	*We need to make sure they're involved from the moment the patient gets their diagnosis... It's also better for the patient to have clear goals from the get-go. HCP12, HCP, Female, 28, Jordan*<br><br>*It also varies greatly depending on the patient. Some people's only priority is to not be in pain, while others can tolerate the pain but want to be able to walk again. Every patient focuses on something. Others want to not be too drowsy from the medication so they can spend time with the people around them, even if it means lower doses and more pain. They'd rather not waste their final days…and just want to be aware enough to socialize with the people around them. HCP06, HCP, Female, 31, Jordan*<br><br>*I want to live long enough to achieve my goal. I want to be an Associate Professor. I'm trying to do it. I will be satisfied if I can write just one line on my manuscript each day. This is my goal. My life will be happy if I achieve this goal. 1005, Patient, Male, 52, Thailand*
• Supporting self-care management	*It is very important for the patient to be his own doctor. PKH0017, Patient, Male, 49, Jordan*<br><br>*I set up three things we need to discuss and educate the patient and their family on them. Patient education helps the patient get ready to go home without having to constantly return because they don't know how to deal with certain things. HCP12, HCP, Female, 28, Jordan*<br><br>*Confidence is critical, but that confidence must come from education. So that you can teach people that they can manage their own body. That the fundamental thing is you managing your body, don't let your body manage you. PAT01-001, Patient, Male, 67, South Africa*<br><br>*What we encounter, some of our COPD patients they actually do not know how to use their pumps. So what we normally do, we have a session where I teach them how to use the pumps the correct way because they will come and tell me the pumps is not working for me. PROF07-003, HCP, Female, 55, South Africa*<br><br>*If I get tired, I observe my body, whether it is swelling or not. I adjust the dosage of medication that the doctor taught me. If I don't feel better, I will come here. I live alone so I have to help myself as much as I can. 1001, Patient, Male, 44, Thailand*

	I think we need to be aware of how patients can take care of themselves at home. We normally…ask them to monitor their own weight at home. However, some patients do not have a weighing machine at home and some patients don't know how to measure the amount of urine. It is difficult for patients to follow all the advice. Some of them don't have a blood pressure monitoring device. It would be great if we could have these devices for patients to borrow. 3004, HCP, Female, 25, Thailand
• Care plans can be accessed by patients and healthcare providers	*We could help…in the way the doctors communicate with you when they change your treatment, yeah to tell you that they are changing a certain thing. PAT03-002, Patient, Male, 56, South Africa*<br><br>*I don't ask. It's all right there in the lab results, my hemoglobin is 11.5 and it should be between 12.5 and 17, it's not that bad if it decreased a little. PAL0039, Patient, Male, 71, Jordan*
**P4. Integration of care**	[See subdomain quotations]
**Communication and information sharing for coordination and continuity of care across the continuum of care**	*They round the floor when they hand over and tell each other what every patient needs. Handover was good. CAL0003, Caregiver, Female, 48, Jordan*<br><br>*One problem is that the imaging reports are still not available on the software. On the software, my latest imaging is the one I had in May, whereas the most recent on I did was in August. So, if anyone takes a look on my file, they won't see the liver mets. Every time I go, they ask for a printed report. This was inconvenient for me because when I went to the surgeon to see if I was eligible for surgery, I had to update them on my situation every time I went there. PKH0011, Patient, Female, 44, Jordan*<br><br>*The nurse that takes the bloods she disappears - I think she even went home that day and the management did not know she is gone. They had to find somebody who is in charge and he had to bring somebody else on board. CAR07-001, Caregiver, Female, 69, South Africa*<br><br>*They work together very well and step-by step. We have met a nurse, a doctor and a pharmacist and we have found that they transfer patient's information among their team very well. They don't ask the same questions. 2007, Caregiver, Female, 62, Thailand*<br><br>*We don't have to waste our time since the treatment process runs smoothly. The doctor tells us about the disease, gives us the whole picture, and explains how to take medication whereas the pharmacist talks to us about the medications and their side effects in details. A nurse will advise us how to take care of the patient, how to choose food and about making an appointment. 2006, Caregiver, Female, 62, Thailand*
• Between healthcare providers	*We have a morning round with the coordinator, social workers, and the PharmD, where we all complete an assessment of the patient and discuss it afterwards. We then decide what to do with the patient or what to change. If the patient mentions something to me after the round, I'll let the doctor know and they'll make a decision, let the resident know, then let everyone in the service know. HCP12, HCP, Female, 28, Jordan*<br><br>*Nurses have a meeting on Tuesdays and Thursdays when we discuss a number of patients for the upcoming week and which doctors are in charge of the patients. If we have a new patient coming into our clinic, we will then cooperate in keeping all staff informed. 3012, HCP, Female, 20, Thailand*<br><br>*Before we refer to another unit, we contact that unit to inform them about our patient's condition and other relevant information. We also advise our patient on the process and where they should contact. We will then scan patient's information and send it to that unit. We can track whether our patient really goes to that unit and gets treatment or not on our computer. 3004, HCP, Female, 25, Thailand*

• Referrals to specialist	*Unfortunately, we don't have a lot of communication among the other departments. We write referrals for the patients and send them to other departments to figure it out, which isn't okay, so we just admit them and ask for consults instead. HCP18, HCP, Male, 54, Jordan*
• Discharge communication	*There's a system in place. The patient knows when their appointments are and gets updated via text. HCP06, HCP, Female, 31, Jordan*<br><br>*We start preparing the patient from the day of admission to the discharge …we get the proper mattress and generator and whatever else needs to be ready at their homes. By the time the patient is discharged, the patient's home is supposed to be ready for care. We have the patient take a 24 hour leave from the hospital and if the patient's family can manage the NG tubes, PEG tubes, the Foley's catheter, the wound etc. HCP8, HCP, Male, 30, Jordan*<br><br>*They mark your file COPD…and that's it. No follow-up or whatever. We're envisioning per our earlier discussions some kind of liaison type of situation. I think that, that thing should be put on to that process immediately! After the very first visit. And I don't think it should be voluntarily either. Uhm, because you'll get a whole lot of denial. PAT01-001, Patient, Male, 67, South Africa*<br><br>*I had lost follow up from my nephrologist for 3 months because I was admitted to another hospital. He sent me a message via LINE to ask why I didn't go to see him. Just doing that made me glad. He could remember me and he cared about me. If a doctor can smoothly approach me, it will make me feel okay. 1005, Patient, Male, 52, Thailand*<br><br>*I also advise them on how to do telemonitoring which is a method we use to monitor patients' symptoms via telephone. Normally, a doctor will order us to do telemonitoring with patients after they visit our clinic. We will call patients and ask them about their heart failure symptoms. 3004, HCP, Female, 25, Thailand*
• Providing access to information and resources	*[Data corresponded to this domain are listed under more specific relevant domains]*
**<u>O1. Access to care</u>**	*It's hard to get the ambulance or when she has a regular follow up the ambulance won't come, her husband puts her in the car, the nurse carries her and I hold the chair and we cram in the car, she gets in a lot of pain.* CKH0015, Caregiver, Female, 67, Jordan<br><br>*My transport is my main thing I need. So, I just leave it like that because sometimes, maybe I get a COPD attack in the night and then I phone the ambulance, they do not come. Like the other time I was waiting for eight hours, till the morning and I had to ask my neighbour to come bring me here. PAT03-001, Patient, Male, 42, South Africa*<br><br>Many patients travelled from remote provinces…those whose appointments had been scheduled for many consecutive days had to stay in Bangkok for several nights. When they stayed in an unfamiliar accommodation, they couldn't sleep thus making them have fatigue and higher blood pressure. 3012, HCP, Female, 20, Thailand
**O1a. Timely access to care**	*[See subdomain quotations]*
• Wait times for referrals to see specialists, to receive a consult	*From my experience with working in different government or public hospitals, about 50% of patients are affected by delayed care due to the medical personnel. This results in patients visiting multiple physicians until they are in stage 4, and not a single one of those doctors would think of malignancy. For example, all of the colon cancer patients go and see about 20 doctors who misdiagnose the patients with IBS. HCP13, HCP, Male, 35, Jordan*

	The result of the biopsy was supposed to come out in two weeks, but it came out after two months, and by that time, there was already a new tumour, around 12cm in size. All of that was in Libya. PKH0021, Patient, Female, 26, Jordan<br><br>*I used to see a doctor at a local hospital. At that time, I still did not know that I had heart disease…The doctor told me that I might have heart disease but that the hospital did not have enough medical devices nor an echocardiogram. He gave me some medicines, but I did not get any better. 1006, Patient, Female, 58, Thailand*
• During consult, to be seen at emergency community care, pre-hospital, hospital, post-hospital; secondary care; time for patient care	*I wish they would take into consideration that some patients travel a long way to take the medication and they have families to return to, so if they could hurry up with the preparation process or the imaging. PAL0037, Patient, Female, 45, Jordan*<br><br>*It takes long, and sometimes I would be in so much pain I can't wait. [In the Emergency Room] I would wait for two or three hours until my turn comes.* PKH0030, Patient, Female, 33, Jordan<br><br>*How long must the people sit here? For instance, they say your appointment is 7 o' clock then they call you 11 o'clock, 12 o'clock. You sit here the whole day. Nothing to eat, nothing to drink. CAR03-009, Caregiver, Female, 61, South Africa*<br><br>*By the time they get to us they just want their pump. Because they have been at the doctor the whole day and they have been at the pharmacy probably for an hour, so no, they do not talk to us. PROF01-009, HCP, Female, 42, South Africa*<br><br>*When we were receiving treatment from other places, we didn't feel good. We had to wait three hours to see the doctor, but the examination lasted for only five minutes. So, I don't think that was appropriate for patients with serious conditions. 2009, Caregiver, Female, 45, Thailand*<br><br>*It would be better if we could have a unit that is in charge of referring patients with heart failure so that the process would be quicker and we could admit more patients. 3006, HCP, Male, 24, Thailand*
**O1b. Care availability**	[See subdomain quotations]
• Availability of healthcare practitioners during and outside of working hours	*If I call them at 3 in the morning and I tell them my complaints, they don't get annoyed. This is what a real doctor is, not those whom when you call start telling you that they're in the clinic and that you should come to them only on clinic time.* PKH0013, Patient, Male, 68, Jordan<br><br>*We barely see [the doctor], where is he? He is supposed to follow up with her. She was in the hospital, she was your patient, go see her. Go console her family, tell them…in these 13-15 days I haven't seen the doctor once.* CKH0015, Caregiver, Female, 67, Jordan<br><br>*Sometimes you come here and just for example, there are just one or two doctors and just for example where there is supposed to be six doctors…and now you do not see a doctor and then you see a nurse or someone like that to attend to you. They must just try to improve that. PAT07-001, Patient, Male, 58, South Africa*<br><br>*The main thing I would say, I have never consulted the doctor here for about two years…Because there is never a doctor that you consult. You just go to that Club. PAT07-001, Patient, Male, 69, South Africa*<br><br>*They may need the information from us and a doctor. However, it is quite difficult to contact a doctor, so they mainly ask us. 3004, HCP, Female, 25, Thailand*

**O1c. Financial burden**	*Finances are one of the main aspects we try to secure for these patients because this also affects their mental health. We see many depressed patients as a result of financial struggles. HCP1, HCP, Male, 41, Jordan*<br><br>*Financially it is quite a strain because they cannot work, so they struggle. A few of them get grants but there is not enough money for transport, there is not enough money to have good nutrition, they do not have good heating. PROF01-002, HCP, Male, 45, South Africa*<br><br>*We have got a lot of impact since we have to stay in Bangkok. The cost of living is so expensive here. 1002, Patient, Female, 22, Thailand*
• Affordability of care including complimentary care and therapies, dental, pharmacare, ambulance	*They started charging me 15 JD for the hormonal therapy. So that's 25 JD for every trip to the hospital. It's a burden. This year two of my sons are in college, so that's an extra burden. PK0012, Patient, Female, 50, Jordan*<br><br>*This cycle is probably the last one I am gonna take because I can't afford any more. I can barely get my children bread…I went to UNHCR and they told me funding was stopped especially for those who came in 2014 like us.* PAL0037, Patient, Female, 45, Jordan<br><br>*Basically I need to have that R200 bucks as a spare in case I am gonna need to Taxify or Uber or I am going to need to have for an emergency of which it's very difficult when you having only one source ye income…So, what you will find now it puts a strain on what do we cut from grocery list? CAR04-002, Caregiver, Female, 26, South Africa*<br><br>*I can use the Universal Coverage Scheme at this hospital. I always come here with my family so I don't feel stressed. 1006, Patient, Female, 58, Thailand*<br><br>*The doctor is considering if an additional medication should be prescribed but I have to pay an additional 1,500 baht per month approximately. It is a diabetes medication which would improve my condition; I think that it would be nice but I'm making up my mind. 1007, Patient, Male, 60, Thailand*
**<u>O2. Patient-Reported Outcomes (PROs)</u>**	[See subdomain quotations]
**O2a. Patient-Reported Outcomes Measures (PROMs)**	[See subdomain quotations]
• Health-Related Quality of Life	*The most common symptom we see is pain, which greatly affects the quality of life of the patient. Pain could limit their activity and keep them in bed. HCP3, HCP, Male, 40, Jordan*<br><br>*Improving quality of life, that is something I think doctors do not think about much. That is actually something that the family addresses often. They will come and tell you: when we go to the shop my husband cannot walk so fast. PROF04-002, HCP, Female, 29, South Africa*<br><br>*Everyone wants their symptoms to go away, but that is impossible. Being less tired and able to live normally without worsening symptoms is good enough. 1012, Patient, Male, 72, Thailand*
• Symptoms	*All I care about is for the pain to go away. I don't really care about the disease. PKH0011, Patient, Female, 44, Jordan*<br><br>*My concern is that he is in a lot of pain at night and I mean the medication does not help. CAR01-003, Caregiver, Female, 60, South Africa*

	She is coughing up phlegm and then she is vomiting, she gets nauseous and wants to vomit and she do not get a appetite as well. CAR03-003, Caregiver, Female, 29, South Africa<br><br>*My symptoms are sometimes good and sometimes bad. It depends on my diet. If I eat what the doctor has told me not to or drink too much water, I will get a relapse. 1014, Patient, Male, 41, Thailand*
• Functionality	*Even if I still have the disease, I want to wake up in the morning, make coffee, I don't have to cook, I can fix myself something simple, move around the house. I want to reduce the pain so I can be around [my family] again. Go out with them on weekends. PKH0011, Patient, Female, 44, Jordan*<br><br>*This disease…it takes away your self will and it does it in an insidious way in that no matter how determined you might be, you can't physically do x, y or z because you can't breathe, and if you can't breathe you can't do anything. PAT01-001, Patient, Male, 67, South Africa*<br><br>The patients want to get better steadily until they can go about their normal daily life and go back to work. At the beginning of treatment, the patients get very worried but later after continuous treatment, they would see positive results in which their condition would improve if they took good care of themselves. Even though they wouldn't completely recover, they got better steadily till they could live their normal daily life and work. They then looked happier. 3008, HCP, Female, 42, Thailand
• Psychosocial outcomes	*We have a duty to do a psychological assessment and support. This is something we can definitely improve. HCP10, HCP, Female, 40, Jordan*<br><br>*But then the social aspects and the psychological aspect is quite important in terms of what support do they have, are they able to do things, do they have somebody to care for them at home, what is it and how does their disease affect their life? PROF01-003, HCP, Female, 31, South Africa*<br><br>*[More corresponding quotations listed within domain 'P2b. Providing supportive care: Sensitivity to emotional/psychosocial needs]*
**O2b. Patient-Reported Experiences (PREMs)**	*I said to [the supervisor], listen, you know what, these people are really rude…He said, no, no you must come, come and he took me to another guy, he is the supervisor and he wanted to call them and know who they are and I said to him, no, do not worry but…he was typing, he said, he is going to send them an email now, right through. He does not tolerate that kind of thing. PAT01-005, Patient, Male, 69, South Africa*<br><br>*We want to come here because the doctor here is a specialist, and my mother trusts the doctors here. If the symptoms worsen in the future, we believe the treatment will be available here. If she goes to a hospital near home, she will not receive the treatment she should get. 2008, Caregiver, Female, 35, Thailand*
• Recommendation or rating of hospital, healthcare provider	*I heard that this department is the best department at Al-Bashir, so I couldn't go see any other doctor. PAL0040, Patient, Female, 43, Jordan*
• Assessment of care, including appropriateness and acceptability of care (competency, knowledge, skills of staff)	*My patients seem to be happy, and their families seem to be happy. But it is not ideal yet. We ought to be assessing in a less sot of biased way…Thinking about the inpatient, we can do a patient satisfaction survey on discharge, and we could give one for the patient, and one for the family, because we have duties to both in palliative care. We could do this in all setting i.e., inpatients, outpatients and homecare patients. HCP10, HCP, Female, 40, Jordan*

**O2c. Patient-Reported Adverse Outcomes (PRAOs)**	*I feel my condition keeps worsening…I have complications every day. PKH0036, Patient, Female, 31, Jordan*<br><br>*They tend to get an exacerbation because the weather has changed, it has become more windy, it has become colder. There are other environmental triggers that are playing into their disease and the small reactive component of their chronic obstructive airways disease. PROF01-005, HCP, Male, 32, South Africa*<br><br>*Because it makes me very sick. Chemo destroyed my life. It takes me the entire time between two cycles to get better. PKH0030, Patient, Female, 33, Jordan*<br><br>*And if the patient says that this medication does not dingises with my body it makes me feel nauseous, it makes me feel – it just does not make me feel right, I feel that they must listen to that and try to change the medication. Not just give the same medication again. CAR03-003, Caregiver, Female, 29, South Africa*<br><br>*Before I got better, the doctor adjusted the dose of medicines several times. It took me a while to get better after adjusting medicines. I got some side effects like dizziness, so I had to see the doctor very often. 1006, Patient, Female, 58, Thailand*
• New or worsening symptoms	*He deteriorated after the biopsy and his movement became difficult and his speech became slurred, he also couldn't control urination, this was all because there were fluids in his head. CAL0013, Caregiver, Male, 55, Jordan*<br><br>*The experiences that we face in the clinics are basically, even though we have a set clinic on Wednesdays for COPD and asthma patients, they can come in at any time. So there is always somebody in the passage battling to breathe or something so it has become the norm in the clinics. PROF07-001, HCP, Female, 51, South Africa*<br><br>*He was very tired from the beginning and got more and more tired. 2005, Caregiver, Female, 50, Thailand*
• Unanticipated visits to healthcare facilities	*Because we come for the whole week when it is wrong. You understand, and then I have to come here, which is not the right thing. CAR04-001, Caregiver, Female, 48, South Africa*<br><br>*At that time I suddenly couldn't breathe, lost my strength, and had swelling so I told my relatives to call a taxi to take me to hospital. I was admitted to the ICU and a doctor spoke to my relatives about life-threatening pulmonary oedema. 1011, Patient, Male, 61, Thailand*
• Death	
