# Supplementary material for: Developing a global practice-based framework of person-centred care from primary data: a cross-national qualitative study with patients, caregivers and healthcare professionals
Source: BMJ Glob Health. 2022 Jul 13;7(7):e008843. doi: 10.1136/bmjgh-2022-008843 (PMC9280875; doi:10.1136/bmjgh-2022-008843)
Supplement: online supplemental file 8 [file bmjgh-2022-008843supp008.pdf]

Supplementary Table 3. Illustrative participant quotations for inductive themes

Inductive overarching person-centred care theme		Illustrative quotations
**Collectivism**		
**Interdependence of wellbeing**<br>• **Patient worries about impact of their illness or death on loved ones**<br>• **Not wanting to burden family members**	*1*	*Interviewer: Are you afraid of death? Patient: No, I am afraid I might leave [my children]. Interviewer: Isn't there anyone who could take care of them? Patient: It won't be like their own mother. PKH0030, Patient, Female, 33, Jordan*
	2	*Some people want to die because they believe their family would be better off without them. HCP14, HCP, Male, 40, Jordan*
	3	*Interviewer: What is important to you? Participant: Important is my health, my children. Interviewer: What about them? Participant: Is only the one child that has a problem. She is a problem and now she is sick. PAT07-005, Patient, Female, 45, South Africa*
	4	*Their decisions depend on their relatives, not them. Patients will choose to do things that would not be a burden to their relatives. For example, patients who have to come to hospital often don't want to come because they don't want their relatives to take leave from work. 3007, HCP, Female, 27, Thailand*
**Dependence on others and benefits of a support system and positive relationships on wellbeing**<br>• **Encourage diagnosis**<br>• **Family members help each other to share care load**<br>• **Support each other psychologically**<br>• **Share health knowledge**<br>• **Guide patient through healthcare system**<br>• **Help to enhance mobility and functionality**<br>• **Financial support**	*5*	*Now my sister helps me and takes to the bathroom, bathes me and helps me get dressed as I became dependent. I used to make my own bed or do dishes, but I do these things much less frequently now. I rarely enter the kitchen or cook now. My daughters have to do these things now. PAL0004, Patient, Female, 46, Jordan*
	6	*Interviewer: Did anyone encourage you to get [the lump] checked? Patient: My husband. PKH0026, Patient, Female, 49, Jordan*
	7	*My kids help me…My son who's in Britain sold his car and clear his bank account for me, and my daughter and husband did the same. They keep telling me not to worry, so financially it is managed. PKH0034, Patient, Female, 61, Jordan*
	8	*These patients really need quite a lot of support because they are so dependent on those who are around them and what is happening around them. You know, it upsets all aspects of their lives because they cannot look after themselves. PROF07-004, HCP, Male, 50, South Africa*
	9	*I always come here with my family so I don't feel stressed. My daughter can manage everything for me…where to contact and where to go. 1006, Patient, Female, 58, Thailand*
	10	*My friend is a cardiologist. If my symptoms get worse before the next follow-up visit, I will call my friend to ask how to cope with my symptoms before seeing a doctor. 1005, Patient, Male, 52, Thailand*
	11	*If my mom is not at home, I will have to wipe him down and take care of his meals. My mom and I usually help each other to take care of him. 2005, Caregiver, Female, 50, Thailand*
**The importance of relationships**<br>• **The value of strong relationships for company, psychological and spiritual support**<br>• **Effects of illness on patient's close relationships**<br>• **Disagreements between patient and family member about health lifestyle**	*12*	*I have a group of friends who would pull me out of the hole every time I felt depressed and they keep telling me what a fighter and a survivor I am. Support groups are very important for cancer patients. PKH0017, Patient, Male, 49, Jordan*
	13	*Psychological problems include depression and social withdrawal, they feel socially rejected – or not rejected – they feel like they can't engage with society as they'd like to, resulting in self-isolation. HCP13, HCP, Male, 35, Jordan*
	14	*[My husband] has always been sweet, I am a bit rough with him nowadays to be honest [because of] the disease and everything. My life is not as it was before, my living, our intimate relationship…he tries to get close to me but I keep drifting away [because I am] in pain, tired and everything. PKH0036, Patient, Female, 31, Jordan*
	15	*The people that I work with actually they are my counsellors…my colleagues, they do that for me and they come to my mother, counselling, talking with her so, she can just open up. So, my colleagues are actually my pillar of emotional needs that I need now, they are actually there for me now. CAR03-008 Caregiver, Female, 39, South Africa*
	16	

• **Risk of social isolation**	17	*The patients that I know off at the moment, the interesting thing is their families are quite frustrated with them. And I think they get a lifetime of dad being rude, irritable, maybe contributing to often quiet, sad broken homes. PROF04-001, HCP, Female, 39, South Africa*
	18	*These patients generally have no relatives to look after them thus get neglected. Those who live by themselves will therefore become stressed; this makes their condition become aggravated. They are upset about nobody looking after them, so they are prone to poor diet restriction. 3010, HCP, Female, 43, Thailand*
		There's an elderly lady who told me that she enjoyed the company of the doctor and nurse on their home visit. 3008, HCP, Female, 42, Thailand
**Changed familial roles**	19	*I think I have grown too fast, maybe 20 years older than I should be, I am responsible for everything in the house, my father feels like a son to me, I would be measuring his temperature or blood pressure. CAL0005, Caregiver, Female, 27, Jordan*
	20	*"I cannot be able to work in the brother's situation. I used to work in the previous years before he got sick" Carer, 04-001, South Africa*
	21	*Some patients are head of the family thus shoulder lots of expectations and don't want to be a burden on their family. 3010, HCP, Female, 43, Thailand*
**Value of condition-specific patient and family support groups**	22	*We have a big hall where we gather the patients there to communicate, talk and to express their problems, pains and everything. I will evoke the idea and give them the space to discuss it, for instance, the first one will talk about his/ her problem then another one will share some words. I think they are the only people who can understand their circumstances more than the doctor just because they had this experience with all these obstacles and they know very well the challenges. HCP16, HCP, Male, 35, Jordan*
	23	*It would be nice if we could help each other. For us too to help. To have something that we do in order to show other people that even us, we can do this, we just need to persevere. Yes, for us to persevere and not lose hope. PAT04-002, Patient, Male, 38, South Africa*
	24	*It is very necessary to talk about this with other patients who have medical conditions. It would be good if we could talk to someone with knowledge. I used to watch a TV program that let the elderly talk to one another about their health. I could use the knowledge I got from their experiences to take care of myself. 1004, Patient, Male, 81, Thailand*
**Educating the household, social network and community about the condition**	25	*I want the family to understand the patient's conditions, like the healthcare provider does… Because the patient is anxious and worried and might have certain social issues from the pressure they get from the family and the people around them. This creates a charged social environment where the family become aggressive and starts to neglect the patient. HCP4, HCP, Female, 31, Jordan*
	26	*"We need to educate them also on that…because the family smokes in the house and that is one of the triggers" HCP, 07-003, South Africa*
	27	*It would be good if they can tell us whether we are taking care of him in the right way or not, such as how we set our house facilities and the food that we cook for the patient. 2006, Caregiver, Female, 62, Thailand*
**Bringing services into the local community or home**		
**Patient not wanting to be admitted/spend long periods in hospital** • **Desire for home visits**	28	*I would tell him I don't want to be admitted…because I had recently moved to a new place and I want to stay with my children, and I would ask him if I can just take the chemo and go home. PKH0030, Patient, Female, 33, Jordan*
	29	*It is mentally better for me and her to leave the hospital…Maybe they could arrange a home visit by a committee every once in a while. It helps her, because no matter what, they provide better care than we do. CKH0019, Caregiver, Female, 40, Jordan*
	30	*I think if they say we are struggling at home, we have the home-based care systems that the sisters come around but it is unfortunately also not an everyday support system. PROF01-008, HCP, Female, 26, South Africa*
	31	*Half of the time they actually need to come into hospital to come and collect their meds. That really is a dream wish, for us to have their meds delivered at home. PROF07-001, HCP, Female, 51, South Africa*
	32	*A home visit would be great. We would feel that the team and the hospital care for us. It would encourage us to go on. 2005, Caregiver, Female, 50, Thailand*
	33	*Home visits will help us better follow up with patients' symptoms. Relatives of bedridden patients will have trouble bringing the patients to the hospital. If we can pay a home visit, it would be convenient for them. 3009, HCP, Female, 21, Thailand*

**Drawing on other human resources in community e.g., health promoters or volunteers; support from community or religious organisations**	*34*<br><br><br>*35*<br><br>*36*<br><br>*37*	*If we can get a multi-pronged approach, where we have social workers, homecare-based carers, we have home visits, we have social workers able to actually go to the communities and see what is available in the community and upskill those people, that will be very useful. PROF01-002, HCP, Male, 45, South Africa*<br><br>*Maybe there is something to be done about that where health promoters are the ones who give more education. PROF03-003, HCP, Female, 35, South Africa*<br><br>*A village volunteer's visit would be nice to help patients. If the patient's condition worsens, there would be someone to inform the relatives to take the patient to the hospital. 1010, Patient, Female, 60, Thailand*<br><br>*In other provinces, there are home visit teams called "village house volunteers". The volunteers help people to take care of patients at home. However, I think it would be too tiring and difficult for the team here since they already have a lot of patients seeing them at the hospital. But there are primary care units and local hospitals in the countryside, and they don't have too many patients. They can see patients in the morning and do a home visit in the afternoon with the village house volunteers. 2002, Caregiver, Female, 53, Thailand*
**Creating a health-promoting home environment**	*38*<br><br><br>*39*	*We start preparing the patient from the day of admission to the discharge. We get the equipment and with donations from the social worker, we get the proper mattress and generator and whatever else needs to be ready at their homes. HCP8, HCP, Male, 30, Jordan*<br><br>*I would like them to give some advice on home arrangement. We'd like them to see whether the toilet is appropriate for the patient or not. If they see our house, they may give some further relevant advice, especially how we can prevent her from falling and how to maintain cleanliness and hygiene. 2002, Caregiver, Female, 53, Thailand*
**Traditional and complimentary medicine**	40<br><br>41<br><br>42	
**Equity and non-discrimination**		
**Wish to be treated with equal respect and care irrespective of socioeconomic status**<br><br>• **Ensure services are accessible and understandable by persons of all socioeconomic backgrounds and ages**	*43*<br><br><br><br>*44*<br><br><br>*45*<br><br>*46*<br><br><br>*47*	*KHCC is two steps away from where I live, but they assigned her to Al-Bashir, I feel KHCC is only for those who are well-connected…why is it only available for certain people? CAL0016, Caregiver, Female, 28, Jordan*<br>*Private hospital sector has turned into a business…It's not a humane profession anymore. I remember the late Dr X giving cash to patients who can't afford to buy medications.*<br><br>*Sometimes I think it is how much you earn and how much money you get in your bank account…I think if you have lots of money, people will help you with respect. If you got less, they treat you like nothing sometimes. CAR03-008, Caregiver, Female, 39, South Africa*<br><br>*If the doctor talks about other things with the patients, it will slow down the patient's examination. Other patients are waiting, so I have to be considerate. 1010, Patient, Female, 60, Thailand*<br><br>*[Patients] have different education and socioeconomic backgrounds. They should have some knowledge so that they can understand this kind of service system. I think that doctors, nowadays, have improved in their communication skills so they can talk to patients. 1005, Patient, Male, 52, Thailand*<br><br>*I'm not good at communicating via the Internet like this clinic does as I'm old-school and not good with computers. 1007, Patient, Male, 60, Thailand*
**Underprivileged patients and families in need of additional social support**	*48*<br><br><br>*49*<br><br><br>*50*	*Some of them are very well off and tell us that they don't need any kind of financial aid. Others are in a terrible financial situation and need financial aid and housing, this is where the social worker intervenes to provide them with a hotel suite and compensate them for transportation so they can reach KHCC. HCP7, HCP*<br><br>*We try to be more aware of the social situation they are facing, and the heightened emotional pressure. I have asked the team to take extra care of their caregivers, to check how they are doing, spend some time with them, because they have not got someone else. We need to be the ones to fill that gap. HCP10, HCP, Female, 40, Jordan*<br><br>*I think more emphasis in identifying patients who need more help. PROF01-001, HCP, Female, 30, South Africa*<br>*Some patients require home visit service while some others require equipment support. Some didn't have money to buy a set of scales, so the clinic lent it to them so that they could weigh themselves and evaluate if they had fluid overload or not. 3008, HCP, Female, 42, Thailand*

**Patients and carers wish for most urgent cases to be prioritised**	51	*This is an ER. They're supposed to act quickly. They don't have to stick to the line, they should let in patients who have a more serious condition that others. PKH0016, Patient, Female, 63, Jordan*
	52	*I would say now to attend to the patients that is very sick. They must go first, you know, and things like that. PAT07-002, Patient, Male, 77, South Africa*
	53	*If the doctor talks about other things with the patients, it will slow down the patient's examination. Other patients are waiting, so I have to be considerate. 1010, Patient, Female, 60, Thailand*
**Reported impacts of a pre-existing relationship between patient and HCP**	54	*When I go for the CT scan, the clerk is very nice to me. I tell her she is like a daughter to me and that I want an appointment in a week, and she does book me an appointment after a week while others might get their appointment booked after two or three months. Sweet talk makes everything easier. PAL0043, Patient, Male, 50, Jordan*
	55	*Some patients are given an appointment a month away. If you know people in the hospital, they can give you an appointment two weeks away. PAL0010, Patient, Male, 65, Jordan*
	56	*I would say I am at an advantage because I know someone who knows someone. So, when I come to the clinic it's about who I know. That will determine whether or not I will be seen or I will be assisted. CAR04-002, Caregiver, X, 26, South Africa*
	57	*It is quite a relationship unlike other patients, you just sort of see them coming in through the triage room and you already know what they need. And you sort of just, let me help you, let me put you in your seat, let us start your neb, let us make you feel better. And they almost become like little VIPs to you. You do not want them to wait. PROF01-007, HCP, Female, 29, South Africa*
	58	*It was taking a long time before the date of the echocardiogram. I had a younger sister working at another provincial hospital in a nearby province, so I went for a check-up at that hospital. 1013, Patient, Male, 50, Thailand*
**Some non-nationals experiencing high financial burden to receive healthcare**	59	*There isn't any discrimination, but they do feel as if they'd have better access to certain services if they were in their country. Some services also aren't available to non-Jordanians… I feel like this is more like injustice. That's how I would feel if I was in a strange country and they told me I couldn't have something or I had to wait for a certain service and only the basic were covered. I would feel the world isn't fair. HCP06, HCP, Female, 31, Jordan*
	60	*The law here states that foreigners are charged more than citizens, so it is not discrimination, it is just the law. CAL0017, Caregiver, Male, 41, Jordan*
	61	*To have health insurance you need to be a Jordanian Citizen or have a national number. As for the Syrian refugees, there are some organizations that cover their treatment like "AlOun" or Caritas. The greatest issue is with Palestinians from Gaza. They can't get healthcare. HCP19, HCP, Male, 55, Jordan*
**Carefulness required when communicating with people of diverse ethnicities and nationalities**<br>• **Some patients report feeling 'better understood' by HCPs of their own ethnicity/language**	62	*There's a lot of socialization during the visits. You're discussing things with someone from a different culture and you both have your questions. HCP06, HCP, Female, 31, Jordan*
	63	*I like the Afrikaans doctors because they understand me better. [And I can speak] my language, yes. So, I cannot say that the foreign doctors are incapable of doing that. They are capable of doing their job but sometimes you just feel like they do not examine you nicely because you do not tell them. PAT03-001, Patient, Male, 42, South Africa*
	64	*I also met a patient who was discouraged…I am from the same province as they are, so I spoke the local language with them…. The doctor told him to avoid high-sodium foods. But our local foods are high in sodium, so I told the patient that we understood…We recommended that he avoid local foods and he listened to us. If we are from the same area as the patient, we understand them better. 3011, HCP, Female, 25, Thailand*
**Sex and gender impacts a) communication dynamic between clinicians and patients or family members b) impacts of serious illness on a person's life**	65	*[The doctor] asked me to call my brother so that he could speak to him. When I called my brother…the doctor took the phone from me and started walking away. When I saw him doing that I started following him and trying to get closer to him, then he entered a room like this one and closed the door. I tried to get in but he wouldn't let me. PAL0009, Patient, Female, 42, Jordan*
	66	*I come from a masculine family, as all of my 7 brothers are older than me. They had all the power and us the girls, we had no opinion. PKH0012, Patient, Female, 50, Jordan*
	67	*If the patient is female, then it's usually her husband [who makes care decisions]. If the husband is dead, then it's the oldest son whether it's regarding treatments, resuscitation, or hospital procedures. HCP3, HCP, Male, 40, Jordan*
	68	

	69	*Another thing that upsets me is that I don't get my period anymore, so it feels like my life is over…I really like kids but now even if I decided to get married, I won't be able to have kids. Nobody is gonna want a woman with cancer, let's be realistic. PKH0020, Patient, Female, 42, Jordan*<br><br>*He took me to [an infertility doctor], and he started running tests and procedures, it turned out I had a problem in my tubes. Suddenly he moved to a house near his mom, and she told him he should get re-married…his mother said, "This pomegranate tree is not yielding any fruits, it is ok to cut it down now". PKH0049, Patient, Female, 56, Jordan*
**Shame and stigma**	70<br><br><br>71<br><br><br>72<br><br>73	*At that time, it was known as "that disease" because it affected more people than it does today, and it affected the social status of the family because it has a genetic element, add to that people were afraid it would be contagious, so it was difficult to deal with it outside a medical context. PKH0017, Patient, Male, 49, Jordan*<br><br>*There is a culture of shame towards psychological care, but some families understand that the patient has gotten to the point where they need this help and it's as important as the rest of their treatment. HCP18, HCP, Male, 54, Jordan*<br><br>*Some people like to gossip. Even in church also…they say, hey, this guy, he is coughing too much, maybe he got TB. He is going to spread it to everyone. PAT03-001, Patient, Male, 42, South Africa*<br><br>*She does not want to live since she has urinary and faecal incontinence. She did not want us to clean for her but we told her that when we were young she had helped us a lot and cleaned for us. 2002, Caregiver, Female, 53, Thailand*
**Variation in care quality received across clinicians and across public vs private and rural vs urban health facilities**	74<br><br><br>75<br><br><br>76	*The thing with public hospitals is that you have to wait for your turn, they do tens of surgeries a day…They [should] increase their capacity, the thing is the private sector doctor charges ten times the doctors here. That's why public hospitals don't hold on to the good doctors and pay them what they deserve, so they flee. PAL0003, Patient, Male, 71, Jordan*<br><br>*At least you know that with private care, you happy, but then with public care we do not have a positive thinking towards…public healthcare…A private doctor where I pay or alternately, go to a bigger hospital such as Groote Schuur. Because then there I will get more information than what I am getting from the smaller hospitals. Most of the time. CAR04-002, Caregiver, X, 26, South Africa*<br><br>*I used to see a doctor in a local hospital and I found that the medical devices, medical skills and clinical experiences of the team were limited. 2002, Caregiver, Female, 53, Thailand*
**Context of limited resources**		
**Limited financial and human resources for health services impacts ability to provide PCC**<br><br>• **Need for adequate clinicians for patient demand**	77<br><br><br>78<br><br>79<br><br><br>80	*We don't always have the appropriate medications, but I use what's available, sometimes I have to resort to psychological support. As a result, I end up taking it upon myself to provide these services including coordination, palliation, radiotherapy, chemotherapy, and spiritual support as well. I end up feeling unsatisfied with myself thinking that I haven't done enough, when in reality I can't.* HCP13, HCP, Male, 35, Jordan<br><br>*We have very qualified experts, but not enough of them, so they can barely cover the patients on the floor. HCP06, HCP, Female, 31, Jordan*<br><br>*In terms of pharmaceutical supply, there are efforts being made to try and prevent stock outs of medicines but nonetheless, that's sometimes an issue we face. For things like Formoterol, Salmeterol, the long-acting agents that we try and put our patients on at some point they tend to be more expensive and so, you cannot purchase this easily. PROF01-005, HCP, Male, 32, South Africa*<br><br>*There would be some policy, financial, personnel, and equipment issues [to provide better care]. Not all hospitals have the necessary resources. 2009, Caregiver, Female, 45, Thailand*
**Limited personal resources of patients and families – a need to address the social determinants of health**<br><br>• **Poor housing and employment conditions contributing to poor health and wellbeing**	81<br><br>82<br><br><br>83	*Financially, I want [my children] to be covered, but my financial status is below zero. We rented two rooms in a house that leaks when it rains and has no heating. PAL0037, Patient, Female, 45, Jordan*<br><br>*Our housing system is not alright, some of these patients are coming from informal settlements. So, it's crowded…so even if moss I don't smoke, if next door there is smoking, and all of we live here I can be easily affected by the next person. Do you understand! So, proper housing that is something us as health care workers that we do not have control over that. PROF03-002, HCP, Female, 28, South Africa*<br><br>*I worked in construction…on the demolition…There is lots of dust. So, I changed on that one and went to Unite Plumbing now …Even there we must drill and turn all things. It is dusty moss there. PAT03-004, Patient, Male, 59, South Africa*

• **Patients using health financial assistance or selling therapeutic equipment to cover basic living expenses**	*84*	*Some of the patients sell their pumps to get money and then they come back to the facility, tight chest and we need to give them another pump.” PROF 07-003, HCP, Female, 55, South Africa*
	85	*You try to explain, ok you have got this social problem…maybe this person is going to be able to counsel you and try to help you find ways of dealing with the situation. And people will just tell you that, “I don’t need to talk. All I need is this and that. I need to work. I need employment. I need food. PROF03-003, HCP, Female, 35, South Africa*
	86	*We should know about personal information that might affect patients’ condition such as their jobs because certain jobs can trigger a relapse. 3009, HCP, Female, 21, Thailand*
**Material realities impose constraints on patient ability to engage or adhere**	*87*	*I took 17 sessions of radiotherapy and then I stopped the treatment for financial reasons. PAL0006, Patient, Male, 68, Jordan*
	88	*People are going to say you have to eat healthy and then a lot of people are going to say but how are we going to eat healthy because it is reality you have to eat what you have…nowadays living healthy is very expensive. CAR03-011, Caregiver, X, 42, South Africa*
	89	*Some patients have to take medicines that are not covered by the Universal Health Coverage Scheme. Some of them used to ask us whether they could stop taking that medicine or not because it was so expensive. 3006, HCP, Male, 24, Thailand*
**Adjusted preferences and expectations**	*90*<br>*100*	*Patient’s satisfaction rate is excellent because as I told you we serve a category of people that are mostly livening a simple lifestyle, they don’t require luxurious treatment, they need core simple services that can simply satisfy them and make them happy, such as, doctors, nurses, pharmacy and scans. HCP16, HCP, Male, 35, Jordan*
	101	*You are constantly seeing a new doctor…every time you find yourself you have to answer questions from scratch about medical history which can get annoying, but then you are not going to be annoyed because now you are getting a free service. CAR04-002, Caregiver, X, 26, South Africa*
		I think [the service] is already good. Any inconvenience would result from my own problem. My house is far away, so I have a problem with taking a leave.1013, Patient, Male, 50, Thailand
**Healthcare workforce wellbeing**		
**Overstretched and under-staffed**	*102*	*It’s also exhausting to see 15 palliative care patients on the same day. You need to rest in between patients and recharge so you can provide the best service. HCP12, HCP, Female, 28, Jordan*
	103	*I feel [the nurses] are not comfortable because of the load, they’re not as patient when you talk to them...Nurses play a big role in the patient’s comfort. Feeling comfortable is a big part of healthcare…I think they should cut them some slack. When you increase the staff, you relieve some of the pressure. I don’t know much about their working hours or salaries, and I don’t know if that affects their attitude…Maybe they feel they’re giving too much and still unappreciated.* PKH0018, Patient, Male, 57, Jordan
	104	*Sometimes we give more than we take. My salary is 320 JD and I get 100 JD incentives every 3 months. I worked 100 times worth that salary for 10 years at that hospital. I worked nights for 8 years and the hospital is 15KM away from my house.* CKH0009, Caregiver (and HCP nurse), Female, 44, Jordan
	105	*I just feel that a person brought his/her problem from the house…Now s/he is out of control. There is exhaustion. The pressure is high. CAR04-001 Caregiver, 48, South Africa*
	106	*Right now [the nurses] are not working at ease, they are under pressure because they have to make that target before they go home. They go home at 4 o’ clock you see. Who says they are going to make it and then the patients start getting rude to them. It is not their fault. CAR03-004, Caregiver, Male, 57. South Africa*
	107	*I do understand that doctors there deal with a lot of patients and nurses see patients every day, this can cause them stress. ID 1007, Patient, Male, 60, Thailand*
	108	*I used to give patients my personal Line contact in case they needed a medical advice. It turned out to be too much for me. They were not considerate. When I could not help, I got criticism from them.* ID3007, HCP, Female, 27, Thailand

**Psychological challenges of working with end-of-life patients**<br><br>• **HCPs questioned the feasibility of engaging in psychological support**	*109*	*The first thing we need to do for someone starting in the department is tell them about the kind of patients they'll encounter so they don't get as depressed as I did. Sometimes you might see the families cry and start to cry with them. Sometimes, you need to prepare yourself emotionally and be involved in the round and see how the doctors deal with the patients… It would be nice if someone from the psychiatry department who constantly deals with patients to also see the doctors…I would like to hear about the experiences of my colleagues in the same field. Did they experience the same things I did? Are my feelings normal? Was I affected more than normal? Emotional support is the most important thing we as healthcare providers require. HCP4, HCP, Female, 31, Jordan*
	110	*I need to debrief with somebody, I need to speak to somebody and then also, when I have had a really bad day then I would need to exercise to get rid of all of that emotions. PROF01-008, HCP, Female, 26, South Africa*
	111	*Even if the psychological support existed in a more formal in-depth way, I am not sure how well it would be utilised because just there is work to be done and sometimes I will just carry on because I would rather get home at a normal home going time than have other things that I do even if those things in the long term are for my own health. PROF01-005, HCP, Male, 32, South Africa*
	112	*I think some staff may need to see a psychiatrist in order to vent their feelings so that they can smile when they see patients. I wish they could look back and see that their works can be very useful for patients and how much they can help other people. If they are aware of these they might be more friendly to patients. But if they don't have a chance to cope with their feelings, they may only focus on their workload. ID 2005, Caregiver, Female, 50, Thailand*
